# Impact of SNPs interplay across the locus of *MBL2*, between MBL and Dectin-1 gene, on women’s risk of developing recurrent vulvovaginal infections

**DOI:** 10.1186/s13578-019-0300-4

**Published:** 2019-05-07

**Authors:** Namarta Kalia, Jatinder Singh, Sujata Sharma, Manpreet Kaur

**Affiliations:** 1 Department of Molecular Biology & Biochemistry, Guru Nanak Dev University, Amritsar, India; 2 Department of Obstetrics & Gynaecology, Bebe Nanki Mother and Child Care Centre, Government Medical College, Amritsar, India; 3 Department of Human Genetics, Guru Nanak Dev University, Amritsar, India

**Keywords:** Additional 5′ variants, 3′ UTR SNP, Innate immunity, Haplotype, Gene–gene interaction, Multifactor dimensionality reduction (MDR), Reproductive infectious diseases

## Abstract

**Background:**

Human mannose binding lectin (MBL) and dendritic cell-associated C-type lectin-1 (Dectin-1) are the two prototypical PRRs of innate immunity, whose direct role in recurrent vulvovaginal infections (RVVI) defense has been defined. Previously, MBL insufficiency was proposed as a possible risk factor for the rapid progression of RVVI while, Dectin-1 was found to be playing an active role in the defense. However, the complete genetic bases for the observed low MBL levels are still lacking as our previous studies in harmony with others demonstrated the un-expected genotype–phenotype patterns. This suggested the presence of unidentified regulatory variants that may modulate sMBL levels and risk of RVVI. Therefore, the present study was designed for more inclusive locus-wide *MBL2* analysis and for the possible non-linear interaction analysis of two PRRs that may impact RVVI susceptibility.

**Methods:**

The present study has extended the previous findings by investigating (1) the role of chosen additional SNPs falling in the 5′ near region relating to sMBL levels and RVVI susceptibility, using polymerase chain reaction-restriction fragment length polymorphism, (2) interactions among SNPs within gene by comprehensive locus-wide haplotype analyses of two *MBL2* blocks, (3) gene–gene interaction analyses between two PRRs, using multifactor dimensionality reduction.

**Results:**

rs11003124**_**G, rs7084554_C, rs36014597_G, and rs11003123_A were observed as the minor alleles in the representative North Indian cohort. RVVI cases and its types showed an appreciably high frequency of C allele, its homozygosity and heterozygosity, explaining the observed dominant mode of inheritance of rs7084554 polymorphism in contributing 1.81 fold risk of RVVI. The rs36014597 polymorphism showed the overdominant mode of inheritance, which further depicts that the carrier of a heterozygous genotype of this polymorphism had more extreme phenotype than either of its homozygous carriers in developing 4.07 fold risk of RVVI. sMBL levels significantly varied for rs11003124, rs36014597 and rs11003123 polymorphisms in bacterial vaginosis, while for rs7084554 polymorphism in mixed infection. Independent analysis of 5′ and 3′ haplotype blocks suggested the risk-modifying effect of all the 5′ additional variants, Y/X secretor polymorphism and 3′-UTR SNP i.e. rs10824792. Combined 5′/3′ haplotype analyses depicted the importance of rs36014597; an additional 5′ variant, Y/X and rs10824792 polymorphisms from both the blocks in regulating sMBL levels and RVVI risk. Three gene–gene interaction models involving uni-variant, bi-variant and tri-variant appeared as significant predictors of RVVI risk with cross-validation consistency of 10/10, 9/10 and 5/10, respectively.

**Conclusions:**

The study presented a low-cost reproducible screening design for additional 5′ variants i.e. rs11003124, rs7084554, rs36014597 and rs11003123 of *MBL2* that can act as markers of susceptibility for RVVI or any other diseases. Two additional 5′ variants of *MBL2* i.e. rs7084554 and rs36014597 were suggested as novel molecular markers that may contribute to RVVI risk by varying sMBL levels. Variants of two blocks were found to have more of a combined effect than the independent effect in modulating RVVI susceptibility and sMBL levels. The study presented weak synergistic interaction between *MBL2* and *CLEC7A* in association with RVVI risk. The preliminary data will establish the foundation for the investigation of within gene and between genes interaction analyses towards RVVI susceptibility.

**Electronic supplementary material:**

The online version of this article (10.1186/s13578-019-0300-4) contains supplementary material, which is available to authorized users.

## Background

Three universal pathological conditions of recurrent vulvovaginal infection (RVVI) are bacterial vaginosis (BV), vulvovaginal candidiasis (VVC) and trichomoniasis [1, 2]. Despite the fine knowledge regarding organismal and non-organismal pathogenesis factors, RVVI remains one of the most enigmatic mucosal problems worldwide due to the presence of 20–30% asymptomatic cases (i.e. healthy women with vaginal microbiota composition same as that of RVVI), some of which also had predisposing non-organismal causes [3]. Moreover, women without any known predisposing factors have also been documented to acquire RVVI [4]. Therefore, identification of causal factors that modulate propensity to RVVI in women is much needed. Mannose binding lectin (MBL), encoded by *MBL2* mapped to 10q21.1 has commonly been referred to as an acute phase protein whose serum levels increases following infections [5]. It is an ideal pattern recognition receptor that binds to specific sugars on pathogen’s surface, consequently causing pathogen removal by complement activation, opsonisation and/or phagocytosis [5]. The serum or vaginal fluid levels of MBL have been assessed in RVVI cases by different studies, suggesting its key involvement in pathogenesis of RVVI [6–13].

The MBL levels have been shown to be determined by single nucleotide polymorphisms (SNPs) in coding and promoter region of *MBL2*. The former includes three SNPs, present in exon 1 at rs5030737 (codon 52), rs1800450 (codon 54) and rs1800451 (codon 57), collectively called as *MBL2* structural variations [14]. The codon 54 and 57 SNPs leads to the substitution of glycine with dicarboxylic acids, while codon 52 leads to the substitution of arginine with cysteine in the collagenous region of a monomeric protein resulting in variant monomers [15, 16]. These variant monomers dramatically affect the circulating levels of higher order functional MBL oligomers [17]. In addition to these, three other promoter region variations i.e. rs11003125 (L/H), rs7096206 (Y/X) and rs7095891 (P/Q) have been functionally authenticated to alter *MBL2* transcription, clearly depicting the importance these genetic variations regarding MBL expression and its circulating levels [16, 18].

The linkage disequilibrium (LD) has been described in the literature between structural and promoter polymorphisms leading to the formation of seven standard haplotypes. These standard haplotypes include HYPA, LYPA, LYQA, LYPB, LXPA, LYQC and HYPD. These haplotypes are commonly referred to as the secretor haplotypes because they regulate sMBL levels [19]. However, additional secretor haplotypes including HXPA, LYQB, HYQA, HYQB, HXQB, LXPB, LXQB, and LYPD have also been reported by various studies in different populations [11, 20–23]. The novel haplotypes have been suggested to be observed, due to genetic heterogeneity between different populations and selective advantage in response to environmental pressures like infections or geographic location [24, 25].

From the past, the structural polymorphisms of *MBL2* remain the centre point of most of the investigations including RVVI. As for instance, the association of *MBL2* structural polymorphisms with RVVI have been documented in different populations [6–8, 26–30]. Our previous study has also elucidated the involvement of standard *MBL2* haplotypes in modulating sMBL levels and RVVI susceptibility [11]. However, our study in harmony with others found an un-expected correlation pattern of genotypes with phenotypes, suggesting the presence of unrecognised regulatory elements of *MBL2* [11, 19, 31]. This implicates the need for further inclusive locus-wide *MBL2* analysis to reveal unrecognised regulatory variants that may modulate sMBL levels and risk of infectious diseases. For this selection of variants can be made based on putative functional effects e.g. altering transcription, translation or miRNA binding.

Our previous study sorted out 12 putative functionally important SNPs of *MBL2* using in silico analysis [32]. From this, evaluation of *MBL2* 3′-UTR SNPs has depicted a novel association of rs10824792 SNP with low sMBL levels and RVVI risk [13]. However, the role of selected SNPs falling in the 5′ near region is still pending to be elucidated. Moreover, high serum levels of Dectin-1, another important innate immune molecule, have also been shown to play an active role in defense against RVVI [12]. Dectin-1, encoded by *CLEC7A* mapped to 12p13.2, is a collaborative PRR that team up with other PRRs via Syk pathway to generate optimal immune responses [33]. Both MBL and Dectin-1 are the essential innate immune components. Therefore to know the relationship between two, when co-activated against same pathogenic stimuli, would be of interest. Our previous investigation suggested the effect of rs3901533 *CLEC7A* SNP in modulating sMBL levels and RVVI susceptibility, suggesting RVVI a multi-factorial phenotype [12]. Though, we did not find any correlation between two proteins, finding the relationship between two genes i.e. *MBL2* and *CLEC7A* is still pending to be elucidated, as the importance of genes before proteins have already been stated.

From this background, the present study was planned to elucidate (1) the role of selected SNPs falling in the 5′ near region relating to sMBL levels and RVVI susceptibility, using a conventional approach (2) interactions among SNPs within gene by comprehensive locus-wide haplotype analyses of *MBL2*, based on the LD pattern obtained across the genomic structure, using genotyped data of *MBL2* variants evaluated in this study and reported previously. (3) gene–gene interaction analyses between two PRRs i.e. MBL and Dectin-1, using multifactor dimensionality reduction method. The preliminary data will establish the foundation for the investigation of within gene and between genes interaction analyses towards RVVI susceptibility.

## Materials and methods

### Study participants

The present study recruited RVVI cases (n = 258, mean age 29.33 years, ± S.D. 8.32) attending Bebe Nanki Mother and Child Care Centre, Department of Obstetrics and Gynaecology, Government Medical College, Amritsar (Pb) and were referred by the gynecologist. These cases were clinically pre-diagnosed with RVVI with minimum 4 documented recurrent experiences in a year, with frequent complaints of smelly discharge, itching, vaginal sores as well as pelvic pain. The controls (n = 203, mean age 29.33 years, ± S.D. 8.17) were matched to cases by age and had, by self-report, no recurrent history of vaginal infection. Participants were excluded if they were known carriers of HIV or any other chronic conditions, under chemotherapy or taking immunosuppressive medications. All the participants provided informed consent in writing. The Institutional Ethics Committee of Guru Nanak Dev University, Amritsar (Punjab), India, approved (Approval no. 06/HG dated 02/01/2015) the study protocol.

### Samples and RVVI categorisation

Two types of samples were collected in the present study i.e. vaginal discharge and peripheral blood samples. Vaginal discharge samples from 200 RVVI cases were carried to the laboratory and subjected to standard diagnostic methods specified in European (IUSTI/WHO) guidelines on vaginal discharge management [34] as reported previously [35]. This categorised 200 RVVI cases into three major categories of RVVI i.e. Bacterial Vaginosis (BV; n = 97), vulvovaginal candidiasis (VVC; n = 62) and Mixed Infections (MI; n = 41) i.e. cases with both BV and VVC. However, 58 RVVI cases could not be processed, hence categorised, as these participants were either menstruating or were not willing to give vaginal samples. The peripheral blood samples (5 ml), collected from all the participants, were further processed for serum and DNA isolation by standard methodology [11–13].

### SNPs selection

In silico analyses demonstrated twelve SNPs of *MBL2* with possible functional consequences to structure and expression of MBL [32]. These twelve putative functional SNPs included rs11003125 (L/H), rs11003124, rs7084554, rs36014597, rs7096206 (Y/X), rs11003123, rs7095891 (P/Q), rs1800450 (codon 54), rs10082466, rs2165813, rs2099903 and rs2099902. Of these, the association of all the SNPs except rs11003124, rs7084554, rs36014597, rs11003123 and rs10082466 with RVVI has been reported previously [11, 13]. Thus, rs11003124, rs7084554, rs36014597 and rs11003123 SNPs except rs10082466 were investigated in the present study to assess their role in RVVI and its types. Hence, all the SNPs of *MBL2* that were prioritised by in silico analyses were validated in relation to RVVI except one 3′UTR SNP i.e. rs10082466. This SNP could not be evaluated as different PCR approaches used for genotyping could not be standardised. Moreover, SNPs flanking rs10082466 SNP were either not functional or of low frequency, so the selection of other regions for sequencing seems expensive, hence not opted.

### Genotype analyses by polymerase chain reaction-restriction fragment length polymorphism (PCR–RFLP)

The present study standardised a simple economical method i.e. PCR–RFLP, for the genotyping of four *MBL2* SNPs including rs11003124, rs7084554, rs36014597, and rs11003123.

#### Primer designing and PCR

NCBI’s Primer-BLAST (https://www.ncbi.nlm.nih.gov/tools/primer-blast/), a freely available online software was used to design primers for PCR amplification. *MBL2* gene sequence (NCBI Reference Sequence: NC_000010.11) flanking the given SNP, was used as an input for the software. The best primer pair was custom-synthesized from Bioserve Biotechnologies (Hyderabad, India). A reaction mixture (20 µl) consisting of template DNA, dNTPs (0.025 mM), *Taq* DNA polymerase (0.3 U) and *Taq* buffer with 15 mM MgCl_2_ (1X) was used for each PCR. Each PCR was performed in a thermal cycler (Applied Biosystems, Life Technologies, USA) with stipulated conditions. The particulars of primers sequence, concentrations of specific primers as well as the amplification conditions intended for all the PCRs have been provided in Table 1. The amplified products were analysed on 1.5% (w/v) EtBr-stained agarose gel (Himedia, India), after electrophoretic separation at 100 V, with gel viewer (Alpha imager, USA).

**Table 1:** PCR-RFLP protocol for the genotyping of *MBL2* additional 5′ variants, PCR primers and conditions

*MBL2* SNPs	Primer type	Primer sequence	Working primer conc. (p mol/µl)	PCR Conditions for all reactions	Product size (bp)
rs11003124^a^	Forward	5′-GCTGGCTTATGCCTGTTAGC-3′	0.05	95 °C for 5 min, followed by 35 cycles (94 °C for 30 s, 53 °C for 30 s, and 72 °C for 45 s) and a final elongation at 72 °C for 5 min	524
	Reverse	5′-CTGCTGAGGTTTCTTAGGGGG-3′	0.05		
rs7084554^a^	Forward	5′-AAGGAGGGGTTCATCTGTGC-3′	0.15		551
	Reverse	5′-TGGGAGGAGGATTCAAGGCA-3′	0.15		
rs36014597^b^	Forward	5′-TCCTGCCAGAAAGTAGAGAGGT-3′	0.15		585
	Reverse	5′-TGGCCTCTAGCTGGGGATTT-3′	0.15		
rs11003123^a^	Forward	5′-CTGGTTCCCCCTTTTCTCCC-3′	0.10		506
	Reverse	5′-TGCACGGTCCCATTTGTTCT-3′	0.10		

^a^Denote SNPs reported at forward strand

^b^Denote SNPs reported at Reverse strand

#### Choice of restriction enzymes and RFLP

**Fig. 1:**
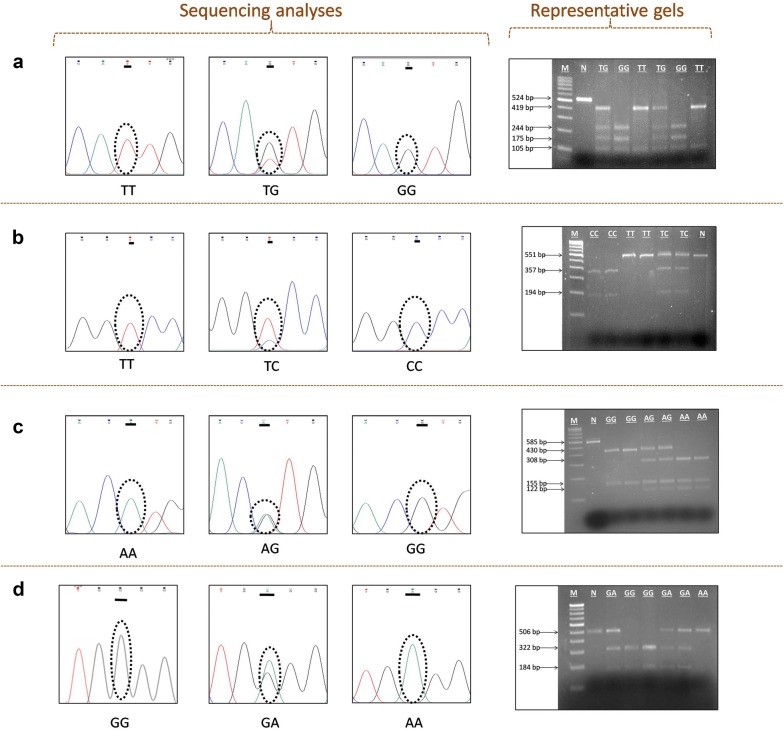
The additional 5′ variants of *MBL2*. Sequencing analyses using forward primer of PCR–RFLP indicating the presence of **a** rs11003124, **b** rs7084554, **c** rs36014597 and **d** rs11003123. Representative agarose gels showing restriction fragment digestion pattern of respective polymorphisms. M: 100 bp DNA ladder. N: negative control

The choice of restriction enzymes to differentiate among variant, wild and heterozygous genotypes for SNPs including rs11003124, rs7084554, rs36014597, and rs11003123 was made with the help of online software NEBcutter v 2.0 (http://nc2.neb.com/NEBcutter2/). Restriction enzyme (IU) along with cut smart NEBuffer (1×) was used for the restriction analysis of amplified PCR product. RFLP conditions along with restriction endonucleases used for the genotyping of each SNP are provided in Table 2. The pattern of restriction digestion was visualised on gel documentation system after electrophoresis on 2.5% (w/v) agarose gel at 100 V. DNA sample of known genotype was used as positive control, while negative control contained all components of restriction digestion mixture except respective restriction enzyme. The PCR products were ascertained by analysing the restriction digestion pattern (Fig. 1). About 10% of the samples with representative genotypes were also confirmed by Sanger sequencing (Fig. 1).

**Table 2:** PCR-RFLP protocol for the genotyping of *MBL2* additional 5’ variants, restriction digestion conditions and expected restriction fragments size for *MBL2* variants

*MBL2* SNP	Enzyme	Restriction site	RFLP conditions	Wild-type homozygotes (bp)	Variant homozygotes (bp)	Heterozygotes (bp)
rs11003124	*TspRI*	5′…NNCASTGNN^▼^…3′ 3′…_▲_NNGTSACNN…5′	22 h at 65 °C	419 + 105	244 + 175 + 105	419 + 244 + 175 + 105
rs7084554	*BanII*	5′…GRGCY^▼^C…3′ 3′…C_▲_YCGRG…5′	4 h at 37 °C	551	357 + 194	551 + 357 + 194
rs36014597	*NLaIII*	5′…CATG^▼^…3′ 3′…_▲_GTAC…5′	4 h at 37 °C	308 + 155 + 122	430 + 155	430 + 308 + 155 + 122
rs11003123	*BseYI*	5′…C^▼^CCAGC…3′ 3′…GGGTC_▲_G…5′	4 h at 37 °C	322 + 184	506	506 + 322 + 184

Single letter code of restriction sites: R = A or G, Y = C or T, N = A or C or G or T, S = C or G

### Haplotype (within a gene) and Gene–gene interaction analyses

For haplotype analyses, SNPs evaluated in the present study along with previously reported SNPs of *MBL2* were considered, to get hold of the entire *MBL2* locus. This include four commonly known secretor polymorphisms [three promoter i.e. rs11003125 (L/H), rs7096206 (Y/X) and rs7095891 (P/Q) and one exonic SNP i.e. rs1800450 (codon 54)], Six 3′UTR polymorphisms [rs10824792, rs2120132, rs2120131, rs2165813, rs2099903 and rs2099902] along with the SNPs evaluated in the present study. The four *MBL2* SNPs i.e. rs11003124, rs7084554, rs36014597, and rs11003123, evaluated in the present study are referred as additional 5′ near gene variants or simply as additional 5′ variants to differentiate them from standard secretor polymorphisms falling in 5′ near gene reported previously. Thus, fourteen SNPs including rs11003125 (L/H), rs11003124, rs7084554, rs36014597, rs7096206 (Y/X), rs11003123, rs7095891 (P/Q), rs1800450 (A/B), rs10824792, rs2120132, rs2120131, rs2165813, rs2099903 and rs2099902 across the *MBL2* locus, from 5′ to 3′ direction were used for haplotype analyses in the present study (Table 3). For gene–gene interactions analyses, total 17 i.e. 14 *MBL2* SNPs and three previously reported *CLEC7A* SNPs i.e. rs3901533, rs11053597 and rs11053593 were considered.

**Table 3:** Features of SNPs used for haplotype analyses across the *MBL2* locus

dbSNP Identifier^a^	SNP position (5′ → 3′)^b^	Chromosome position^b^	Secretor^c^	Region	Nucleotide change
rs11003125	− 618	52,772,254	L/H (aka − 550)	5′ near gene	C/G
rs11003124	− 495	52,772,131		5′ near gene	T/G
rs7084554	− 417	52,772,053		5′ near gene	T/C
rs36014597	− 404	52,772,040		5′ near gene	A/G
rs7096206	− 289	52,771,925	Y/X (aka − 221)	5′ near gene	G/C
rs11003123	− 138	52,771,774		5′ near gene	G/A
rs7095891	− 65	52,771,701	P/Q (aka + 4)	5′ near gene	C/T
rs1800450	+ 161	52,771,475	A/B (codon 54)	Exon1	G/A
rs10824792	+ 4873	52,766,446		3′UTR	T/C
rs2120132	+ 5037	52,766,280		3′UTR	T/C
rs2120131	+ 5058	52,766,258		3′UTR	T/G
rs2165813	+ 5090	52,766,224		3′UTR	G/A
rs2099903	+ 5217	52,766,097		3′UTR	C/A
rs2099902	+ 5225	52,766,089		3′UTR	T/C

^a^NCBI dbSNP database (http://www.ncbi.nlm.nih.gov/SNP)

^b^NCBI contig accession number NT_030059.14 (NCBI build 142, locus ID 4153)

^c^Designation extensively used in literature, is also followed in the present study, these four markers form the ‘secretor haplotypes’

### Serum MBL concentration

The Serum MBL (sMBL) concentration was determined with enzyme-linked immunosorbent assay (Human MBL ELISA kit, Ray Biotech, USA) following the manufacturer’s instructions, as reported previously [11, 13]. Briefly, 100 μl of standard, blank and 4000 fold pre-diluted serum sample was added to the respective wells of microtitre plate, pre-coated with anti-human MBL antibody and incubated for 2.5 h at room temperature. After 3–4 washings with 1× wash buffer, 100 μl of biotinylated anti-human MBL antibody was added and the plate was incubated at room temperature for 1 h. Following incubation, unbound biotin conjugated anti-human antibody was removed by 3–4 washings with 1X wash buffer and 100 μl streptavidin-HRP was added to all the wells, followed by incubation at room temperature for 45 min. After 3–4 washings, 100 μl of 3,3,5,5′-tetramethylbenzidine (TMB) substrate was added and incubated for 30 min at room temperature in the dark. The blue color was developed in proportion to the amount of MBL present in the sample. The reaction was stopped with 50 μl of stop solution (0.2 M H_2_SO_4_) that changes the color from blue to yellow. The intensity of the color was measured at 450 nm by microplate reader (BIO-RAD, iMark™, USA). The assay was calibrated using MBL (25 ng/ml) standard provided in the kit, which was used to prepare different standard dilutions i.e. 8.33, 2.778, 0.926, 0.309, 0.103, 0.034 ng/ml, as instructed in the kit. The assay diluent A, provided in the kit was used as blank and to prepare standard dilutions. All these different standard concentrations were used to obtain MBL standard curve, hence standard equation, from which, the concentration of MBL in the serum samples was determined. The minimum detection sensitivity of the ELISA kit was 0.03 ng/ml.

### Statistical analysis

To achieve a minimum of 80% power for the present study, required sample size was calculated with Genetic Association Study (GAS) power calculator (http://csg.sph.umich.edu/abecasis/gas_power_calculator/), considering assumptions that are 30% countrywide prevalence of abnormal vaginal discharge, 10% MAF, 1.5 odds ratio (OR) and 5% error rate (α = 0.05). Standard adds up was carried out to compute allelic as well as genotypic frequencies of different SNPs in cases and controls. These frequencies were further compared by odds ratio statistics with MedCalc software v 9.3.9.0 (MedCalc Software, Ostend, Belgium). The major allele and its subsequent homozygous genotype were selected as the reference (OR = 1). Deviation of each marker from Hardy–Weinberg equilibrium (HWE) was tested with SNPStats (https://www.snpstats.net/snpstats/start.htm). The best genetic models for each polymorphism was selected based on the smallest Akaike’s Information Criterion (AIC) as well as the Bayesian Information Criterion (BIC) values given by SNPStats. The interpretation of Linkage Disequilibrium (LD) between each polymorphism was done using Haploview v 4.2 (http://www.broad.mit.edu/mpg/haploview/). Haplotypes were inferred with PHASE software v 2.1.1. (http://stephenslab.uchicago.edu/phase/download.html) using genotypic data and for each analysis, the most common haplotype observed in the cohort of the present study was selected as a reference (OR = 1). One-way ANOVA (analyses of variance) with subsequent Tukey’s multiple comparison post hoc test were performed to compare serum biomarkers within group consisting of more than two categories. Comparison of serum biomarkers of cases with respective controls and between two categories of a group was done by Student’s *t* test. Gene–gene interactions analysis was performed with the help of Multifactor Dimensionality Reduction (MDR) software v 3.0.2 (http://www.multifactordimensionalityreduction.org/). Statistical package for social sciences (SPSS) v 16.0 (SPSS Inc., Chicago, IL) was used to perform all these statistical analyses unless mentioned. The p-value ≤ 0.05 were considered to be statistically significant for all data analyses.

## Results

### Genetic analyses of additional 5′ variants in RVVI relative to controls

All the additional 5′ variants conformed to HWE (p > 0.05) except rs11003123 polymorphism. rs11003124**_**G, rs7084554_C, rs36014597_G, and rs11003123_A were found to be minor alleles in North Indian cohort (Table 4). Of all the evaluated additional 5′ variants, allelic and genotypic frequencies significantly varied for rs7084554 and rs36014597 polymorphisms only (Tables 4 and 5). The C allele of rs7084554 was found to be significantly (p = 0.009; OR = 1.54; 95% CI 1.11–2.13) more prevalent in RVVI cases than controls. The frequency of TC heterozygous genotype (p = 0.002) and CC homozygous genotype (p = 0.396) of rs7084554 was high in RVVI cases than controls. Excluding the recessive model of inheritance, all the other models were found to be significant for rs7084554 SNP. However, the dominant genetic model was found to be best, with least AIC = 629.5 and BIC = 641.9 values, depicting that C allele carrier (either in homozygous or heterozygous state) had a greater risk of developing RVVI than non-C carriers (p = 0.002; OR = 1.81; 95% CI 1.22–2.68). Moreover, significantly (p < 0.0001; OR = 2.23; 95% CI 1.62–3.07) higher prevalence of G allele of rs36014597 SNP was observed in RVVI cases comparative to controls. Also, a considerably high prevalence of AG genotype (p < 0.0001) was observed in RVVI cases than controls. The overdominant model of inheritance, with the lowest AIC = 590.5 and BIC = 602.9 values, was found to be best, of all the models that showed significant association with rs36014597 polymorphism. The overdominant model depicts that the carrier of heterozygous genotype will have more extreme phenotype than that of either of its homozygous carriers in developing RVVI risk (p < 0.0001; OR = 4.07; 95% CI 2.69–6.17).

**Table 4:** Distribution of allele frequencies of *MBL2* additional 5′ variants in RVVI cases and controls

Alleles	All subjects	RVVI cases	Controls	RVVI cases vs controls	
	(N = 461)	(N = 258)	(N = 203)	OR (95% CI)	p-value
	Freq (%)	Freq (%)	Freq (%)		
rs11003124					
T	714 (77.44)	400 (77.51)	314 (77.33)	1.00	
G	208 (22.55)	116 (22.48)	92 (22.66)	0.98 (0.72–1.35)	0.94
rs7084554					
T	724 (78.52)	389 (75.38)	335 (82.51)	1.00	
C	198 (21.47)	127 (24.61)	71 (17.48)	1.54 (1.11–2.13)	0.009*
rs36014597					
A	694 (75.27)	356 (68.99)	338 (83.25)	1.00	
G	228 (24.72)	160 (31.00)	68 (16.74)	2.23 (1.62–3.07)	1.2 × 10^−6^**
rs11003123					
G	615 (66.70)	343 (66.47)	272 (66.99)	1.00	
A	307 (33.30)	173 (33.52)	134 (33.01)	1.02 (0.77–1.34)	0.86

*OR* odds ratio, *CI* confidence intervals

* Indicates significant p values (p < 0.01)

** Indicates highly significant values (p < 0.001)

**Table 5:** Distribution of genotypic frequencies along with inheritance models of *MBL2* 5′ additional variants in RVVI cases and controls

Genetic models	Genotype	VVI cases	Controls	OR (95% CI)	p-value	AIC	BIC
		(N = 258)	(N = 203)				
		Freq (%)	Freq (%)				
rs11003124							
Codominant	T/T	155 (60.11)	125 (61.60)	1.00			
	T/G	90 (34.92)	64 (31.50)	1.14 (0.76–1.69)	0.53	639.4	655.9
	G/G	13 (5.00)	14 (6.90)	0.75 (0.34–1.65)	0.58		
Dominant	T/T	155 (60.10)	125 (61.60)	1.00			
	T/G-G/G	103 (39.91)	78 (38.41)	1.07 (0.73–1.56)	0.74	638.4	650.8
Recessive	T/T-T/G	245 (95.23)	189 (93.12)	1.00			
	G/G	13 (5.00)	14 (6.90)	0.72 (0.33–1.56)	0.4	637.8	650.2
Overdominant	T/T-G/G	168 (65.10)	139 (68.50)	1.00			
	T/G	90 (34.90)	64 (31.50)	1.17 (0.79–1.73)	0.45	637.9	650.3
Log-additive	–	–	–	0.99 (0.73–1.34)	0.95	638.5	650.9
rs7084554							
Codominant	T/T	147 (56.97)	143 (70.42)	1.00			
	T/C	95 (36.82)	49 (24.11)	1.90 (1.25–2.89)	0.002*	631	647.6
	C/C	16 (6.21)	11 (5.42)	1.41 (0.63–3.15)	0.396		
Dominant	T/T	147 (57.21)	143 (70.41)	1.00			
	T/C-C/C	111 (43.12)	60 (29.60)	1.81 (1.22–2.68)	0.002*	*629.5*	*641.9*
Recessive	T/T-T/C	242 (93.80)	192 (94.60)	1.00			
	C/C	16 (6.21)	11 (5.41)	1.15 (0.52–2.55)	0.72	638.4	650.8
Overdominant	T/T-C/C	163 (63.21)	154 (75.90)	1.00			
	T/C	95 (36.81)	49 (24.12)	1.85 (1.22–2.79)	0.003*	629.7	642.1
Log-additive	–	–	–	1.50 (1.09–2.07)	0.01*	632	644.4
rs36014597							
Codominant	A/A	110 (42.60)	147 (72.41)	1.00			
	A/G	136 (52.70)	44 (21.70)	4.20 (2.75–6.41)	1.08 × 10^−10^**	592	608.5
	G/G	12 (4.70)	12 (5.90)	1.33 (0.57–3.08)	0.497		
Dominant	A/A	110 (42.60)	147 (72.41)	1.00			
	A/G-G/G	148 (57.42)	56 (27.60)	3.61 (2.42–5.38)	8.8 × 10^−10^**	596	608.4
Recessive	A/A-A/G	246 (95.31)	191 (94.12)	1.00			
	G/G	12(4.70)	12 (5.90)	0.77 (0.34–1.77)	0.54	638.1	650.5
Overdominant	A/A-G/G	122 (47.31)	159 (78.32)	1.00			
	A/G	136 (52.70)	44 (21.70)	4.07 (2.69–6.17)	1.1 × 10^−10^**	*590.5*	*602.9*
Log-additive	–	–	–	2.42(1.72–3.41)	1.2 × 10^−6^**	610.8	623.2
rs11003123							
Codominant	G/G	100 (38.81)	84 (41.42)	1.00			
	G/A	143 (55.41)	104 (51.22)	1.16(0.79–1.70)	0.46	639.5	656
	A/A	15 (5.82)	15 (7.41)	0.84 (0.39–1.82)	0.61		
Dominant	G/G	100 (38.81)	84 (41.42)	1.00			
	G/A-A/A	158 (61.22)	119 (58.61)	1.12 (0.77–1.62)	0.57	638.2	650.6
Recessive	G/G-G/A	243 (94.22)	188 (92.61)	1.00			
	A/A	15 (5.81)	15 (7.40)	0.77 (0.37–1.62)	0.5	638	650.4
Overdominant	G/G-A/A	115 (44.61)	99 (48.80)	1.00			
	G/A	143 (55.41)	104 (51.21)	1.18 (0.82–1.71)	0.37	637.7	650.1
Log-additive	–	–	–	1.03 (0.76–1.40)	0.85	638.5	650.9

Italics values indicate low AIC/BIC value

*AIC* Akaike’s Information Criterion, *BIC* Bayesian Information Criterion

* Indicates (p ≤ 0.01)

** Indicates (*p *≤ 0.001)

**Table 6:** *MBL2* additional 5′ variant’s distribution and comparison of genotypes and alleles in RVVI types with controls

	No. (%) of controls	No. (%) of VVI types			Genotype comparison					
					BV vs HC		VVC vs HC		MI vs HC	
	HC (N = 203)	BV (n = 97)	VVC (n = 62)	MI (n = 41)	OR (95% CI)	p-value	OR (95% CI)	p-value	OR (95% CI)	p-value
rs11003124										
Genotypes										
TT	125 (61.51)	64 (65.91)	36 (58.12)	23 (56.12)	1		1		1	
TG	64 (31.52)	27 (27.82)	26 (41.91)	15 (36.51)	0.82 (0.47–1.41)	0.48	1.41(0.78–2.53)	0.25	1.27 (0.62–2.60)	0.50
GG	14 (6.81)	6 (6.11)	0 (0.00)	3 (7.31)	0.83 (0.30–2.28)	0.72	–	NA	1.16 (0.30–4.37)	0.82
Alleles										
T	314 (77.31)	155 (79.81)	98 (79.12)	61 (74.31)	1		1		1	
G	92 (22.62)	39 (20.12)	26 (20.91)	21 (25.62)	0.85 (0.56–1.30)	0.47	0.90 (0.55–1.47)	0.69	1.17 (0.67–2.03)	0.56
rs7084554										
Genotypes										
TT	143 (70.40)	59 (60.82)	35 (56.41)	21 (51.21)	1		1		1	
TC	49 (24.10)	28 (28.81)	27 (43.52)	17 (41.42)	1.38 (0.79–2.41)	0.24	2.25 (1.23 4.09)	0.007**	2.36 (1.15–4.83)	0.01**
CC	11 (5.41)	10 (10.30)	0 (0.00)	3 (7.30)	2.20 (0.88–5.46)	0.08	–	NA	1.85 (0.47–7.20)	0.37
Alleles										
T	335 (82.50)	146 (75.21)	97 (78.21)	59 (71.90)	1		1		1	
C	71 (17.40)	48 (24.72)	27 (21.72)	23 (28.01)	1.55 (1.02–2.34)	0.03*	1.31 (0.79–2.15)	0.28	1.83 (1.06–3.17)	0.02*
rs36014597										
Genotypes										
AA	147 (72.40)	41 (42.21)	29 (46.77)	18 (36.51)	1		1		1	
AG	44 (21.61)	50 (51.51)	33 (53.21)	19 (46.32)	4.07 (2.39–6.94)	3.7 × 10^−7^***	3.80(2.08–6.94)	1.7 × 10^−5^***	3.52 (1.70–7.29)	0.0007***
GG	12 (5.91)	6 (6.12)	0 (0.00)	4 (9.70)	1.79 (0.63–5.06)	0.27	–	NA	2.72 (0.79–9.34)	0.111
Alleles										
A	338 (83.20)	132 (68.12)	81 (65.31)	49 (59.70)	1		1		1	
G	68 (16.71)	62 (31.91)	33 (26.62)	27 (32.91)	2.33 (1.56–3.47)	3.6 × 10^−5^***	2.02(1.25–3.27)	0.004**	2.73 (1.60–4.68)	0.0002***
rs11003123										
Genotypes										
GG	84 (41.31)	36 (37.11)	26 (41.91)	16 (39.01)	1		1		1	
GA	104 (51.22)	53 (54.62)	36 (582)	21 (51.21)	1.18 (0.71–1.98)	0.50	1.11 (0.62–1.99)	0.70	1.06 (0.52–2.15)	0.87
AA	15 (7.38)	8 (8.21)	0 (0.00)	4 (9.71)	1.24 (0.48–3.19)	0.64	–	NA	1.4 (0.41–4.76)	0.59
Alleles										
G	272 (66.91)	125 (64.4)	88 (70.91)	53 (64.62)	1		1		1	
A	134 (33.12)	69 (35.5)	36 (29.12)	29 (35.31)	1.12 (0.78–1.60)	0.53	0.83 (0.53–1.28)	0.40	1.11(0.67–1.82)	0.67

*OR* odds ratio, *CI* confidence intervals

* Indicates significant p values (p ≤ 0.05)

** Indicates high significant values (p ≤ 0.01)

*** Indicates highly significant values (p ≤ 0.001)

### Genetic analyses of additional 5′ variants in RVVI types relative to controls

For rs7084554, significantly higher prevalence of C allele was observed in BV (p = 0.03) and MI (p = 0.02) than controls (Table 6). Heterozygosity of C allele was observed to be appreciably higher in VVC (p = 0.007) and MI (p = 0.01) than controls. For rs36014597 polymorphism, G allele was significantly more prevalent in BV (p = 0.0001), VVC (p = 0.004) and MI cases (p = 0.0002) as compared to controls. Heterozygosity for G allele was observed to considerably more prevalent in BV (p = 0.0001), VVC (p = 0.0001) and MI cases (p = 0.0007) comparative to controls. However, no significant difference in genotypic as well as allelic distribution was observed for rs11003124 and rs11003123 variants. Also, no homozygote of the minor allele of all the additional 5′ variants was observed in VVC.

### Genotype–phenotype association of additional 5′ variants

The stratification of previously measured sMBL levels was made on the basis of the observed genotypes of additional 5′ variants in cases and controls (Table 7). In BV, overall significant difference between genotypic sMBL levels was observed for rs11003124 (p = 0.026), rs36014597 (p = 0.01) and rs11003123 (p = 0.006) polymorphism. Further analysis of rs11003124 polymorphism indicated that TT (p = 0.032) and TG (p = 0.021) genotypes contributed considerably low measured sMBL levels than GG genotype. For rs36014597 polymorphism, AA (p = 0.007) and AG (p = 0.014) genotypes were significantly contributing low sMBL levels comparative to GG genotype. For rs11003123 polymorphism, considerably low sMBL levels were observed for GA (p = 0.01) genotype than AA genotype. In MI, an overall significant difference between genotypic sMBL levels was observed for rs7084554 (p = 0.01) polymorphism only. Further analysis of this polymorphism indicated that sMBL levels contributed by TT genotype were significantly (p = 0.013) different from levels contributed by TC genotype. Data analysis for genotypic sMBL levels in controls, RVVI and VVC revealed no significant difference in sMBL levels of studied 5′ variants. In addition, for the same genotypes, cases were found to have considerably low sMBL levels than controls.

**Table 7:** Distribution of sMBL levels in RVVI, RVVI types and controls, stratified on the basis of *MBL2* additional 5′ variant’s genotypes

Genotypes	Controls		RVVI		p-value (Controls vs. RVVI)^†^	BV		p-value (Controls vs. BV)^†^	VVC		p-value (Controls vs. VVC)^†^	MI		p-value (Controls vs. MI)^†^
	n	Mean ± SD	n	Mean ± SD		n	Mean ± SD		n	Mean ± SD		n	Mean ± SD	
rs11003124														
TT	125	1080.5 ± 465.50	155	744.07 ± 495.75	1.8 × 10^−8^***	64	678.61 ± 390.79^a^	1.5 × 10^−8^***	36	442.98 ± 184.28	2.7 × 10^−24^***	23	634.42 ± 293.56	2.8 × 10^−7^***
TG	64	1049.9 ± 430.30	90	795.44 ± 531.28	0.002**	27	626.25 ± 326.24^b^	1.5 × 10^−5^***	26	458.20 ± 230.82	1.1 × 10^−12^***	15	887.35 ± 648.44	0.368
GG	14	1313.5 ± 520.39	13	962.81 ± 496.91	0.084	6	1111.8 ± 678.54^ab^	0.472	0	0.00 ± 0.00	NA	3	850.39 ± 25.19	0.152
p-value	^††^	0.144	^††^	0.290		^††^	0.026*		^†^	0.774		^††^	0.234	
rs7084554														
TT	143	1112.4 ± 525.32	147	719.73 ± 478.38	1.4 × 10^−10^***	59	624.45 ± 325.85	2.0 × 10^−13^***	35	447.24 ± 207.63	7.3 × 10^−23^***	21	500.00 ± 104.30^f^	9.7 × 10^−25^***
TC	49	1316.7 ± 679.60	95	865.74 ± 602.85	7.7 × 10^−5^***	28	748.64 ± 483.93	2.1 × 10^−4^***	27	452.23 ± 201.68	1.4 × 10^−11^***	17	929.32 ± 665.41^f^	0.046*
CC	11	1069.6 ± 422.36	16	833.60 ± 321.42	0.111	10	797.20 ± 368.89	0.134	0	0.00 ± 0.00	NA	3	850.39 ± 25.19	0.40
p-value	^††^	0.080	^††^	0.097		^††^	0.218		^†^	0.925		^††^	0.01**	
rs36014597														
AA	147	1165.5 ± 577.79	110	720.49 ± 478.04	2.8 × 10^−10^***	41	634.69 ± 302.67^c^	1.1 × 10^−12^***	29	455.00 ± 207.48	1.7 × 10^−21^***	18	669.20 ± 311.55	2.4 × 10^−6^***
AG	44	1049.0 ± 440.84	136	796.61 ± 527.34	0.005**	50	680.18 ± 417.03^d^	7.0 × 10^−5^***	33	444.32 ± 202.85	2.8 × 10^−11^***	19	887.19 ± 640.10	0.325
GG	12	1297.6 ± 530.97	12	899.07 ± 428.27	0.325	6	1164.3 ± 670.40^cd^	0.05*	0	0.00 ± 0.000	NA	4	842.52 ± 430.05	0.145
p-value	^††^	0.293	^††^	0.328		^††^	0.01**		^†^	0.839		^†^	0.416	
rs11003123														
GG	84	1086.4 ± 466.75	100	754.57 ± 508.33	8.7 × 10^−6^***	36	780.92 ± 474.43	0.001***	26	448.89 ± 199.68	1.9 × 10^−16^***	16	646.30 ± 343.61	0.001***
GA	104	1066.1 ± 430.02	143	769.09 ± 512.02	2.6 × 10^−6^***	53	598.29 ± 322.96^e^	3.7 × 10^−12^***	36	449.89 ± 208.86	1.1 × 10^−20^***	21	872.59 ± 607.82	0.177
AA	15	1143.8 ± 549.47	15	856.24 ± 384.30	0.108	8	1062.2 ± 604.69^e^	0.746	0	0.00 ± 0.000	NA	4	896.06 ± 346.68	0.409
p-value	^††^	0.816	^††^	0.767		^††^	0.006**		^†^	0.985		^††^	0.365	

*NA* not applicable

* p ≤ 0.05, ** p ≤ 0.01, *** p ≤ 0.001

^†^Independent t-test for patient vs. control as well as within groups

^††^One Way ANOVA with overall p-value followed by Post Hoc Tukey test that further indicates values with similar letter are significantly different

^a^p = 0.032, ^b^ p = 0.021, ^c^ p = 0.007, ^d^ p = 0.014, ^e^ p = 0.01, ^f^ p = 0.013

Genotypes with n < 3 are not included in statistical analysis

### Linkage disequilibrium analyses

**Fig. 2:**
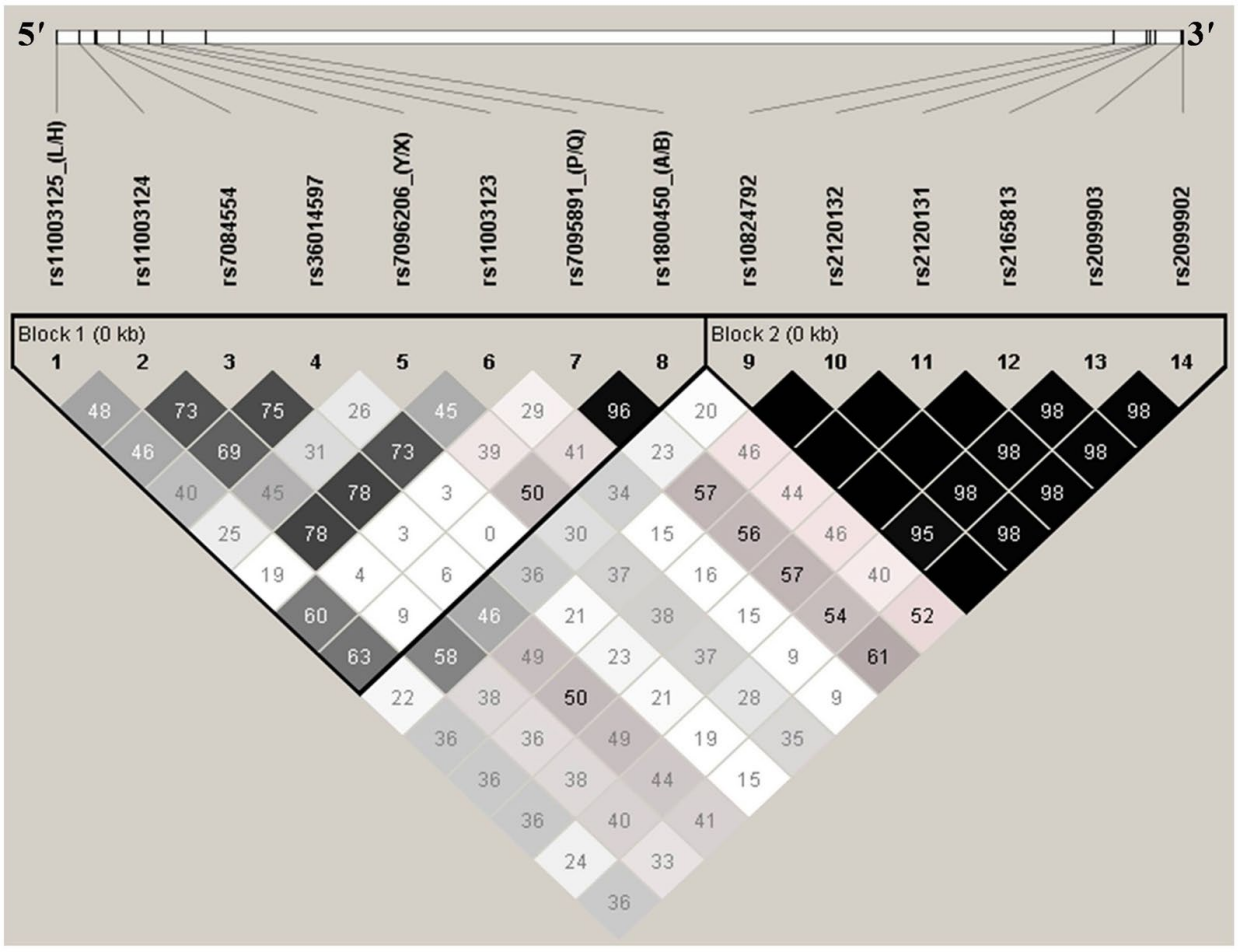
LD pattern of MBL2 variants in all participants. Block 1 represents the 5′ block variants. Block 2 represents the 3′ block variants. D′ is scaled between white diamond: D′ = 0  i.e. complete Linkage equilibrium, shades of grey: 0 < D′ < 1, Black diamond: D′  =  1 i.e. complete LD. Numbers in diamonds are D′-value expressed as a percentile. Black diamond without numbers represents a D′ of 100

The LD pattern of 14 variants across *MBL2* was determined using their genotyped data. As LD is likely to decrease with increase in physical distance, the LD plot with two blocks was observed in the cohort of the present study for *MBL2* (Fig. 2). The secretor polymorphisms along with additional 5′ near gene variants form the 5′ block. The six 3′UTR variants form the 3′ block of *MBL2*. The markers of one block were not in LD with the markers of other. The LD analysis of 5′ block of *MBL2* indicated nearly complete LD between P/Q and codon 54 variants with D′ of 0.96 and thus co-inherited together. SNP pairs including rs11003124/rs11003123, rs7084554/rs11003123, rs7084554/rs36014597, rs11003124/rs7084554 and rs36014597/rs11003123 were in strong LD with D′ of 0.78, 0.78, 0.75, 0.73 and 0.73, respectively. In addition, SNP pairs including rs11003124/rs36014597, LH/AB and LH/PQ showed fairly high LD with D′ of 0.69, 0.63 and 0.6. The LD analysis indicated that all the SNPs of 3′ block are in strong LD with each other and are co-inherited together [13].

### Haplotypes analyses

The LD analyses confirmed two haplotype blocks i.e. 5′ haplotype block and 3′ haplotype block. Both separate and combined analysis of each block was performed. The distribution of 3′ block haplotypes in cases (RVVI and its types) and controls has been reported previously [13]. This analysis showed the presence of three common haplotypes i.e. TTTGCT (3′H-1), CCGAAC (3′H-2) and CTTGCT (3′H-3) with frequency ≥ 0.05 (5%) either in cases or controls. Five rare haplotypes i.e. TTTGAT (3′H-4), CCGACT (3′H-5), CTTGAT (3′H-6), CCTAAT (3′H-7) and CTTGCC (3′H-8) were also observed, but excluded from the previous analysis due to their very low frequency (< 0.05).

#### Distribution of MBL2 5′ block haplotypes

Total fifty-nine 5′ block haplotypes from 5′H-1 to 5′H-59, with frequencies ≥ 0.001 (0.1%) either in cases or controls, were observed in the present study. An overall significant difference (global p = 0.01) were observed in distributions of all the observed 5′ block haplotypes among case–control groups (Additional file 1). Only six haplotypes i.e. HTTAXGQB (5′H-1), HTTAYGQB (5′H-2), LTTAXGQB (5′H-3), LTTAYGPA (5′H-4), LGCGYAPA (5′H-5) and HTTAYAPA (5′H-6) with frequency ≥ 0.05 (5%, either in cases or controls) were observed and are referred as common haplotypes, while the rest haplotypes were considered as rare and thus excluded from the present analysis due to their very low frequency. The case–control comparison of common haplotypes depicted considerably low prevalence of haplotype 5′H-2 in RVVI and MI cases than controls (Table 8). Also, the frequency of haplotype 5′H-4 was observed to be significantly low in RVVI and BV cases than controls. Moreover, the other common haplotypes did not show any statistical differences in their observed frequencies between cases and controls.

**Table 8:** Distribution and comparison of 5′ block haplotypes of *MBL2* in cases and controls

Haplotypes (5′ → 3′)	No. (%) of controls	No. (%) of cases				Haplotype comparison							
		Total RVVI cases	Clinical categories of RVVI			RVVI vs controls		BV vs controls		VVC vs controls		MI vs controls	
	(n = 406)	(n = 516)	BV (n = 194)	VVC (n = 124)	MI (n = 82)	OR (95% CI)	p value	OR (95% CI)	p value	OR (95% CI)	p value	OR (95% CI)	p value
Common													
HTTAXGQB (5′H-1)	52 (12.80)	98 (18.99)	33 (17.01)	20 (16.12)	16 (19.51)	1		1		1		1	
HTTAYGQB (5′H-2)	57 (14.03)	50 (9.68)	19 (9.79)	16 (12.90)	6 (7.31)	0.46 (0.28–0.77)	0.003*	0.52 (0.26–1.03)	0.062	0.72 (0.34–1.55)	0.415	0.34 (0.12–0.94)	0.03*
LTTAXGQB (5′H-3)	39 (9.60)	65 (12.59)	27 (13.91)	17 (13.70)	7 (8.53)	0.88 (0.52–1.48)	0.643	1.09 (0.56–2.10)	0.795	1.13 (0.52–2.44)	0.749	0.58 (0.21–1.55)	0.281
LTTAYGPA (5′H-4)	51 (12.56)	51 (9.88)	12 (6.18)	14 (11.29)	10 (12.19)	0.53 (0.31–0.88)	0.01*	0.37 (0.17–0.79)	0.01*	0.71 (0.32–1.56)	0.399	0.63 (0.26–1.53)	0.315
LGCGYAPA (5′H-5)	24 (5.91)	64 (12.40)	19 (9.79)	14 (11.29)	14 (17.07)	1.41 (0.79–2.52)	0.238	0.55 (0.28–1.09)	0.089	1.51 (0.65–3.50)	0.329	1.89 (0.79–4.50)	0.147
HTTAYAPA (5′H-6)	17 (4.18)	26 (5.03)	11 (5.67)	6 (4.83)	3 (3.65)	0.81 (0.40–1.63)	0.557	1.01 (0.42–2.44)	0.965	0.91 (0.31–2.65)	0.874	0.57 (0.14–2.21)	0.419

Global p-value for case/control haplotype association was 0.01

* p ≤ 0.01

#### Distribution of combined 5′/3′ block haplotypes

The combined haplotype analyses included genotypic data of only 218 participants i.e. 109 cases and 109 controls from the total cohort of the present study, as the same number of participants, were used for the evaluation of 3′ block variants as reported previously [13]. A global significant difference (p = 0.01) in combined 5′/3′ haplotype frequency was observed. Total 86 combined haplotypes, with frequencies ≥ 0.001 either in cases or controls, were observed (Additional file 2). Of these, haplotypes i.e. LTTAYGQB/CCGAAC (5′H-10/3′H-2), LGCGYAPA/TTTGCT (5′H-5/3′H-1), LTTAXGQB/CTTGCT (5′H-3/3′H-3), HTTAXGQB/TTTGCT (5′H-1/3′H-1), HTTAYGPA/TTTGCT (5′H-9/3′H-1), HTTAXGQB/CCGAAC (5′H-1/3′H-2), HTTAXGPA/CTTGCT (5′H-35/3′H-3) and HTTAXGQB/TTTGCT (5′H-60/3′H-1) with frequency ≥ 0.05 (5%, either in cases or controls) were observed and are referred as common haplotypes, while the rest were considered as rare haplotypes and excluded from the present analysis. Considerably higher frequency of 5′H-60/3′H-1 haplotype was observed in BV cases than controls (Table 9). Moreover, the other combined common *MBL2* haplotypes did not show any statistical differences in their observed frequencies between cases and controls.

**Table 9:** Distribution and comparison of combined 5′/3′ haplotypes of *MBL2* in cases and controls

Haplotypes (5′ → 3′)	No. (%) of controls	No. (%) of cases				Haplotype comparison							
		Total RVVI cases	Clinical categories of RVVI			RVVI vs controls		BV vs controls		VVC vs controls		MI vs controls	
	(n = 218)	(n = 218)	BV (n = 112)	VVC (n = 56)	MI (n = 50)	OR (95% CI)	p value	OR (95% CI)	p value	OR (95% CI)	p value	OR (95% CI)	p value
Common													
5′H-10/3′H-2	24 (11.00)	23 (10.55)	9 (8.03)	8 (14.2)	6 (12.00)	1		1		1		1	
5′H-5/3′H-1	12 (5.50)	16 (7.33)	9 (8.03)	3 (5.35)	4 (8.00)	1.39 (0.54–3.56)	0.492	2.00 (0.63–6.34)	0.2395	0.75 (0.16–3.35)	0.706	1.33 (0.31–5.64)	0.695
5′H-3/3′H-3	15 (6.88)	10 (4.58)	4 (3.57)	3 (5.35)	3 (6.00)	0.69 (0.26–1.86)	0.469	0.71 (0.18–2.72)	0.618	0.60 (0.13–2.62)	0.497	0.80 (0.17–3.68)	0.774
5′H-1/3′H-1	14 (6.42)	10 (4.58)	10 (8.92)	0 (0.00)	0 (0.00)	0.74 (0.27–2.01)	0.561	1.90 (0.62–5.81)	0.257	–	NA	–	NA
5′H-9/3′H-1	15 (6.88)	8 (3.66)	4 (3.57)	2 (3.57)	2 (4.00)	0.55 (0.19–1.56)	0.265	0.71 (0.18–2.72)	0.618	0.40 (0.07–2.14)	0.284	0.53 (0.09–2.99)	0.475
5′H-1/3′H-2	9 (4.12)	11 (5.04)	4 (3.57)	5 (8.92	2 (4.00)	1.27 (0.44–3.64)	0.649	1.18 (0.29–4.83)	0.812	1.66 (0.43–06.45)	0.459	0.88 (0.15–5.24)	0.896
5′H-35/3′H-3	4 (1.83)	11 (5.04)	5 (4.46)	3 (5.35)	3 (6.00)	2.86 (0.79–10.31)	0.106	3.33 (0.72–15.26)	0.121	2.25 (0.41–12.28)	0.349	3.00 (0.52–17.15)	0.216
5′H-60/3′H-1	3 (1.37)	11 (5.04)	7 (6.25)	3 (5.35)	1 (2.00)	3.82 (0.94–15.49)	0.060	6.22 (1.31–29.44)	0.021*	3.00 (0.50–17.95)	0.228	1.33 (0.11–15.20)	0.816

Global p-value for case/control haplotype association was 0.01

*NA* not applicable

* p ≤ 0.05; HTTAYGPA (5′H-9), LTTAYGQB (5′H-10), HTTAXGPA (5′H-35), HTTAXGQB (5′H-60)

### Haplotype-phenotype associations

The previously measured sMBL levels were stratified in cases and controls on the basis of independent and combined haplotypes of two blocks. The distribution and comparisons of sMBL levels of 3′ block haplotypes in cases and control groups have been reported previously, which suggested that 3′ block haplotypes may alter sMBL levels and susceptibility to RVVI [13].

#### Distribution of sMBL levels in 5′ block haplotypes

sMBL levels were segregated according to 5′ block common haplotypes in cases and controls (Table 10). In controls, an overall significant difference (p = 0.004) was observed among sMBL of different haplotypes. Further analysis has shown that 5′H-1 (HTTAXGQB; p = 0.002) and 5′H-4 (LTTAYGPA; p = 0.048) haplotypes were conferring significantly low levels comparative to 5′H-2 (HTTAYGQB) haplotype, whereas other haplotypes in controls did not show any significant difference among sMBL levels. In RVVI cases, sMBL levels of different haplotypes showed an overall significant difference (p = 0.008). Further analysis has shown that 5′H-3 (LTTAXGQB) haplotype were accounted for significantly (p = 0.029) low levels than 5′H-2 haplotype, whereas other haplotypes in RVVI cases did not show any significant difference among sMBL levels. In RVVI types i.e. BV, VVC and MI, no overall as well as in particular significant difference was observed among sMBL levels of different haplotypes. Furthermore, significantly (p < 0.05) low sMBL levels were accounted by various haplotypes of RVVI and its types comparative to corresponding haplotypes in controls.

**Table 10:** Distribution of sMBL levels in cases and controls, stratified on the basis of 5′ block haplotypes of *MBL2*

Haplotypes	Controls		RVVI		p-value (controls vs RVVI)^†^	BV		p-value (controls vs BV)^†^	VVC		p-value (controls vs VVC)^†^	MI		p-value (controls vs MI)^†^
	n	Mean ± SD	n	Mean ± SD		n	Mean ± SD		n	Mean ± SD		n	Mean ± SD	
5′H-1	52	929.13 ± 328.26^a^	98	796.14 ± 497.42	0.05*	33	756.96 ± 518.41	0.095	20	495.04 ± 146.90	6.5 × 10^−11^***	16	774.02 ± 512.29	0.268
5′H-2	57	1229.7 ± 457.22^ab^	50	964.66 ± 694.09^c^	0.024*	19	711.44 ± 421.49	4.1 × 10^−5^***	16	568.28 ± 288.36	2.1 × 10^−8^***	6	912.34 ± 720.02	0.132
5′H-3	39	1053.3 ± 521.53	65	666.02 ± 450.61^c^	1.2 × 10^−4^***	27	571.21 ± 222.96	3.8 × 10^−6^***	17	419.29 ± 282.35	3.2 × 10^−7^***	7	701.91 ± 401.10	0.098
5′H-4	51	1001.6 ± 333.61^b^	51	703.54 ± 505.09	0.001***	12	763.35 ± 554.86	0.177	14	372.70 ± 182.16	1.7 × 10^−11^***	10	691.51 ± 367.44	0.01**
5′H-5	24	1075.7 ± 353.79	64	896.52 ± 548.16	0.077	19	883.99 ± 511.06	0.210	14	563.78 ± 384.49	1.8 × 10^−4^***	14	990.78 ± 618.07	0.643
5′H-6	17	1188.7 ± 461.46	26	631.93 ± 248.25	1.5 × 10^−4^***	11	715.82 ± 302.77	0.003**	6	487.56 ± 220.87	1.1 × 10^−4^***	3	663.52 ± 23.84	2.4 × 10^−4^***
p-value^††^		0.004*		0.008*			0.333			0.272			0.697	

* p ≤ 0.05, ** p ≤ 0.01, *** p ≤ 0.001

^†^Independent t-test between patients and controls as well as within groups

^††^One Way ANOVA with overall p-value followed by Post Hoc Tukey test that further indicates values with similar letter are significantly different

^a^p = 0.002, ^b^ p = 0.048, ^c^ p = 0.029

#### Distribution of sMBL levels in combined 5′/3′ block haplotypes

sMBL levels were segregated according to combined 5′/3′ block common haplotypes in cases and controls (Table 11). In controls, only an overall significant difference (p = 0.032) was observed among sMBL of different haplotypes. In RVVI cases, an overall significant difference (p = 0.002) was observed among sMBL of different haplotypes. Further analysis has shown that 5′H-10/3′H-2 (LTTAYGQB/CCGAAC) haplotype accounted for significantly low sMBL levels than 5′H-5/3′H-1 (LGCGYAPA/TTTGCT; p = 0.033) and 5′H-1/3′H-2 (HTTAXGQB/CCGAAC; p = 0.010) haplotypes. Also, 5′H-35/3′H-3 (HTTAXGPA/CTTGCT) haplotype was contributing significantly (p = 0.021) low sMBL levels than 5′H-1/3′H-2 haplotype whereas, no other significant differences were observed. In BV and VVC cases, different haplotypes did not show any in particular significant difference among sMBL levels, though overall significant difference (p = 0.050) was found in BV. In MI cases, an overall significant difference (p = 0.006) was observed among sMBL levels of different haplotypes. Further analysis has shown that 5′H-10/3′H-2 (LTTAYGQB/CCGAAC; p = 0.008) and 5′H-35/3′H-3 (HTTAXGPA/CTTGCT; p = 0.016) haplotypes accounted for significantly low sMBL levels than 5′H-5/3′H-1 (LGCGYAPA/TTTGCT) haplotype. Furthermore, significantly (p < 0.05) low sMBL levels were accounted by various haplotypes of RVVI and its types comparative to respective haplotypes in controls.

**Table 11:** Distribution of sMBL levels in cases and controls, stratified on the basis of combined 5′/3′ block haplotypes of *MBL2*

Haplotypes	Controls		RVVI		p-value (controls vs RVVI)^†^	BV		p-value (controls vs BV)^†^	VVC		p-value (controls vs VVC)^†^	MI		p-value (controls vs MI)^†^
	n	Mean ± SD	n	Mean ± SD		n	Mean ± SD		n	Mean ± SD		n	Mean ± SD	
5′H-10/3′H-2	24	836.70 ± 293.17	23	434.33 ± 190.36^ab^	1.7 × 10^−6^***	9	466.93 ± 253.23	0.002**	8	348.26 ± 113.77	1.9 × 10^−7^***	6	502.68 ± 148.28^d^	0.001***
5′H-5/3′H-1	12	957.48 ± 453.28	16	857.95 ± 549.84^a^	0.614	9	976.38 ± 598.13	0.935	3	296.06 ± 345.25	0.036*	4	1042.5 ± 282.10^d e^	0.733
5′H-3/3′H-3	15	1267.1 ± 418.30	10	714.96 ± 513.21	0.007**	4	792.13 ± 657.76	0.090	3	541.73 ± 635.47	0.02*	3	785.30 ± 260.96	0.076
5′H-1/3′H-1	14	1261.6 ± 612.37	10	795.10 ± 425.08	0.050*	10	795.10 ± 425.08	0.050*	0	0.00 ± 0.00	NA	0	0.00 ± 0.00	NA
5′H-9/3′H-1	15	1228.8 ± 545.30	8	729.92 ± 407.04	0.034*	4	788.98 ± 463.87	0.160	2	796.85 ± 645.86	NA	2	544.88 ± 75.72	NA
5′H-1/3′H-2	9	999.78 ± 162.15	11	969.51 ± 530.29^bc^	0.861	4	1113.4 ± 138.52	0.251	5	934.80 ± 788.63	0.864	2	768.50 ± 311.79	NA
5′H-35/3′H-3	4	1070.9 ± 362.48	11	389.29 ± 194.90^c^	3.6 × 10^−4^***	5	363.78 ± 281.19	0.013*	3	346.46 ± 51.56	0.026*	3	466.14 ± 127.39^e^	0.042*
5′H-60/3′H-1	3	915.49 ± 320.84	11	735.61 ± 320.19	0.405	7	578.27 ± 174.35	0.057	3	576.38 ± 110.23	0.158	1	1159.05 ± 0.00	NA
p-value^††^		0.032*		0.002**			0.050*			0.269			0.006**	

Haplotypes with n < 3 are not included in statistical analysis

* p ≤ 0.05, ** p ≤ 0.01, *** p ≤ 0.001

^†^Independent t-test between patients and controls as well as within groups

^††^One Way ANOVA with overall p-value followed by Post Hoc Tukey test that further indicates values with similar letter are significantly different

^a^p = 0.033, ^b^ p = 0.010, ^c^ p = 0.021, ^d^ p = 0.008, ^e^ p = 0.016

### Gene–gene interaction analyses

**Fig. 3:**
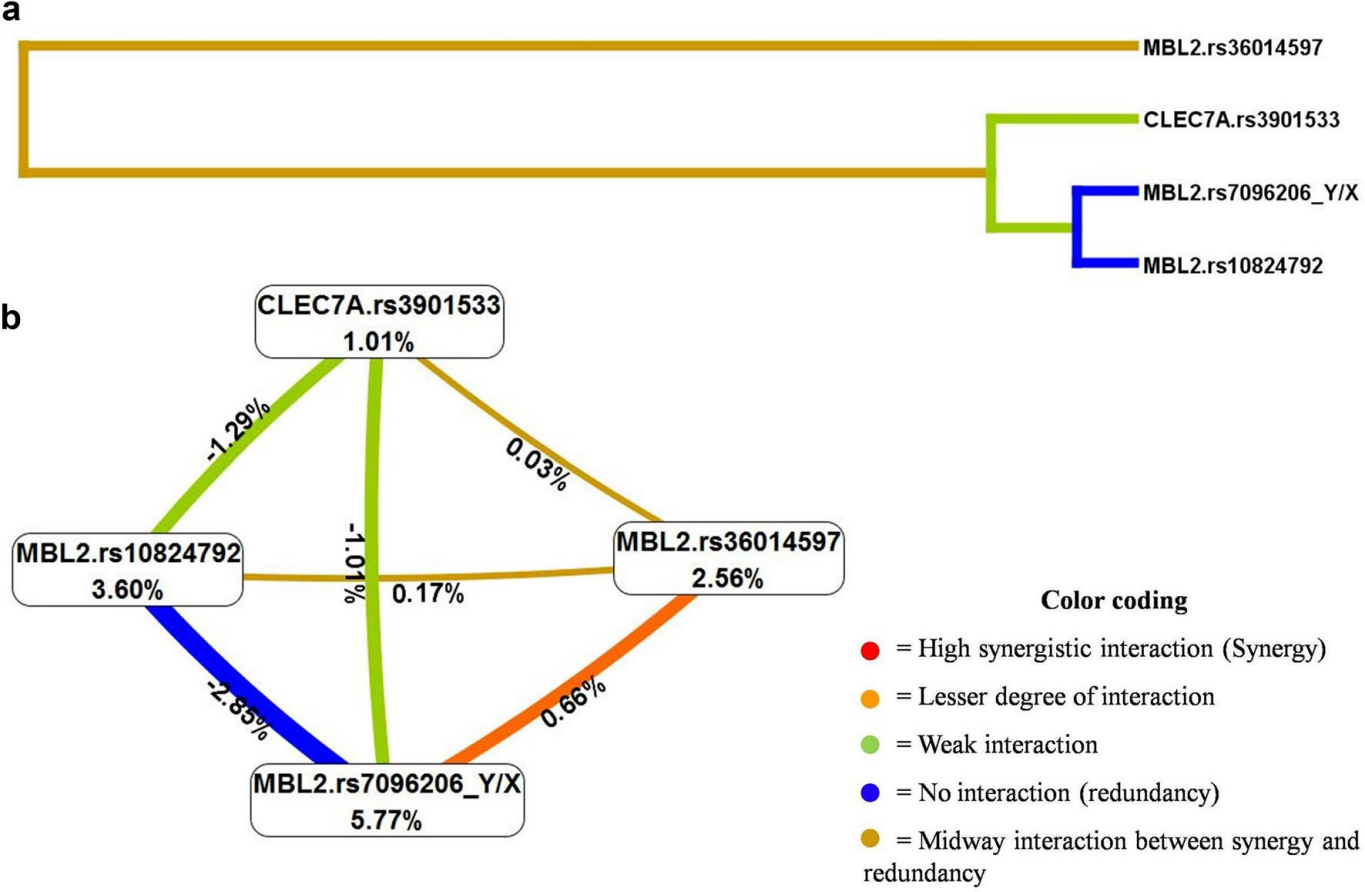
**a** The dendrogram representing the nature of possible interaction between SNPs by MDR analysis. **b** Interaction entropy model by MDR

Multifactor dimensionality reduction (MDR) method was used to study interaction between all the seventeen SNPs of two genes i.e. *MBL2* and *CELC7A.* Three gene–gene interaction models involving uni-variant, bi-variant and tri-variant appeared as significant (p < 0.001) predictors of RVVI risk (Table 12). Among 17 SNPs, one-way interaction model including *MBL2* Y/X polymorphism was found to have maximum cross-validation consistency (CVC) of 10/10 with testing balance accuracy of 63.70%. Two-way interaction model (*MBL2*.rs36014597 and *MBL2*.Y/X) and three-way interaction model (*MBL2*.Y/X, *MBL2*.rs10824792 and *CLEC7A*.rs3901533) also showed association with RVVI risk but had comparatively low CV consistency i.e. 9/10 and 5/10, respectively relative to the uni-variant model. MDR analysis provides dendrogram to interpret the nature of possible interaction between SNPs (Fig. 3a). It was found that *MBL2*.Y/X, *MBL2*.rs10824792 and *CLEC7A*.rs3901533 belonging to one group had shown a weak synergistic interaction in predicting RVVI risk. This group along with *MBL2*.rs36014597 showed an intermediate level of association between synergy and redundancy in predicting RVVI susceptibility. MDR analysis also provides a graphical model of entropy interaction (Fig. 3b). It was found that entropy-based analysis was positive (0.03%) for a pairwise effect of *CLEC7A*.rs3901533 and *MBL2*.rs36014597 indicating synergy while negative for pair *CLEC7A*.rs3901533 and *MBL2*.Y/X (− 1.01%) as well as pair *CLEC7A*.rs3901533 and *MBL2*.rs10824792 (− 1.29%) indicating redundancy towards RVVI susceptibility.

**Table 12:** Multifactor dimensionality reduction (MDR) analysis for the identification of best gene–gene interaction models for RVVI cases and controls

Models	SNPs interaction	Training balance accuracy	Testing balance accuracy	Cross validation consistency	OR (95% CI)	χ^2^ value	p-value
1	*MBL2*.rs7096206 (Y/X)	0.637	0.637	10/10	3.25 (1.84–5.74)	17.15	< 0.0001*
2	*MBL2*.rs36014597, *MBL2*.rs7096206 (Y/X)	0.657	0.623	9/10	5.33 (2.71–10.50)	26.05	< 0.0001*
3	*MBL2*.rs7096206 (Y/X), *MBL2*.rs10824792, *CLEC7A*.rs3901533	0.702	0.637	5/10	5.73 (3.15–10.45)	35.10	< 0.0001*

* Indicates significant at p ≤ 0.001

## Discussion

The present study is the first report presenting the frequency distribution of the four additional 5′ near gene variants of *MBL2* in North Indian cohort, which depicted rs11003124_G, rs7084554_C, rs36014597_G, rs11003123_A to be minor alleles. The distribution of these variants was in agreement with all the different populations of the 1000 Genomes Project (Phase 3) except African population owing to its population substructure, high genetic diversity and less LD between genetic loci (Additional file 3) [36]. The two SNPs i.e. rs7084554 and rs36014597 were found to be significantly predisposing individuals to RVVI and its types, while the other two variants i.e. rs11003124 and rs11003123 was not found to be associated with the disease condition. Significantly higher prevalence of C allele, its homozygosity and heterozygosity were observed in RVVI cases and its types as compared to controls, explaining the observed dominant mode of inheritance of rs7084554 polymorphism that is increasing the risk of RVVI. In addition, the best fit model of rs36014597 polymorphism showed the overdominant mode of inheritance, which further depicts that the carrier of the heterozygous genotype of this polymorphism had more extreme phenotype than either of its homozygous carriers in developing RVVI risk as complemented by the observed results of genotype distribution. The literature search did not reveal any articles that have evaluated the role of select 5′ additional variants of *MBL2* in association with RVVI or its types. However, these polymorphisms have been evaluated in relation to leprosy and malaria with no significant associations [23, 37]. An association of rs11003124 and rs11003123 SNPs have been shown with the increased risk of leprosy and hepatocellular carcinoma in patients with hepatitis B-related cirrhosis, respectively [37, 38].

Linking genotypes of these polymorphisms with phenotypes showed that for the same genotypes, cases accounted significantly low levels than controls. Furthermore, genotypic sMBL levels significantly varied for rs11003124, rs36014597 and rs11003123 polymorphisms in BV, while for rs7084554 polymorphism in MI. However, sMBL levels for these particular loci did not vary as expected from their genetic association analysis, possibly due to the small size of the respective groups, as the study was underpowered for RVVI categories. Therefore, increasing the sample size may ascertain the role of these SNPs in susceptibility to BV and MI. To date, no studies have examined the genotype–phenotype correlation of these polymorphisms. However, a single and recent study depicted no significant difference in genotypic sMBL levels of rs11003124 polymorphism in bakery workers with work-related respiratory symptoms [39]. Thus, of the 14 screened polymorphisms of *MBL2*, the single variant analysis showed five polymorphisms including rs7096206 (Y/X), rs7084554, rs36014597, rs10824792, and rs2099903 have been found to be associated with RVVI risk [11, 13]. For the association studies, the haplotype-based analysis has been suggested to be more influential approach, which can help avoid the risk of misinterpretation of individual SNPs analysis [40]. Therefore, haplotypes were constructed on the basis of linkage disequilibrium analysis as 5′ and 3′ block haplotypes. These blocks were evaluated independently as well as in combination, as it’s the different combinations of amino acids in a polypeptide chain that collectively determine the protein structure and function.

Independent analyses of 5′ block haplotypes have shown a significantly low prevalence of common haplotypes i.e. HTTAYGQB (5′H-2) in RVVI and MI cases as well as haplotype LTTAYGPA (5′H-4) in RVVI and BV cases relative to controls. This depicts that the major alleles (marked by underline) of 5′ additional variants and Y/X secretor polymorphism are collectively conferring protection against RVVI, BV and MI cases. Evaluation of *MBL2* 3′ block haplotypes analyses showed the presence of three common haplotypes i.e. TTTGCT (3′H-1), CCGAAC (3′H-2) and CTTGCT (3′H-3) in RVVI cases and controls. Independent analyses 3′ block haplotypes showed the risk effect of 3′H-3, the haplotype including the minor allele of rs10824792 SNP only in RVVI [13].

Combined 5′/3′ haplotypes analyses have shown significantly high prevalence of 5′H-60/3′H-1 (HTTAXGQB/TTTGCT) haplotype in BV cases than controls, showing X allele as an important marker for conferring risk of disease development. Thus, independent analysis of two blocks suggested the risk-modifying effect of 5′ additional variants, Y/X secretor polymorphism and 3′-UTR SNP i.e. rs10824792. However, complete haplotype analysis depicted only Y/X polymorphism as an important marker for determining disease risk. It also suggested the possibility of unrevealed regulating variants that may be masking the effect of others. Therefore, for further clarification, haplotype-phenotype correlation analysis was carried out.

Independent analyses of 5′ block haplotypes with sMBL levels showed significant difference in sMBL levels of different haplotypes, in controls and RVVI cases. Further analysis depicted, 5′H-1 (HTTAXGQB) haplotype accounted for significantly low levels than 5′H-2 (HTTAYGQB) haplotype in controls. Also, 5′H-3 (LTTAXGQB) haplotype accounted for significantly low levels than 5′H-2 haplotype in RVVI. These results again suggested the contribution of Y/X variant in modulating sMBL levels in line with the above findings of the present study. Moreover, stratification of sMBL levels based on 3′ block haplotypes suggested 3′H-3, the haplotype with the minor allele of rs10824792 SNP only, accounted for significantly low sMBL levels, and RVVI risk [13]. Considering this, it is possible that both 5′ and 3′ haplotypes contribute to sMBL levels, but at this point, it is still tentative whether these blocks have an independent impact on the sMBL levels or they have combined effect.

Therefore, sMBL levels were further correlated with combined 5′/3′ haplotypes. Low sMBL levels were observed in various haplotypes of RVVI and its types as compared to respective haplotypes in controls. Moreover, an overall significant difference was observed among sMBL levels of different haplotypes in controls, RVVI, BV, and MI cases. Analyses in these groups showed that 5′H-10/3′H-2 haplotype having LYQB a low secretor haplotype in 5′ block in combination with the minor allele haplotype of 3′ block was contributing low level than 5′H-5/3′H-1 i.e. haplotype having LYPA a high secretor haplotype in 5′ block and the major allele haplotype of 3′ block in RVVI and MI cases. Thus, the inclusion of additional variants in haplotype analysis solved the previously reported discrepancy where a high secretor haplotype i.e. LYPA accounted for significantly low sMBL levels than low secretor haplotype i.e. LYQB in all cases types and controls [11]. Also, 5′H-35/3′H-3 haplotype having low secretor haplotype ‘HXPA’ and 3′ block haplotype ‘CTTGCT’ with only risk allele ‘C’ of SNP rs10824792 was significantly contributing low sMBL levels than 5′H-5/3′H-1 haplotype with high secretor haplotype ‘LYPA’ and major allele haplotype of 3′ block in MI cases. Thus, correlation of combined haplotypes with sMBL levels signifies the importance of Y/X and rs10824792 polymorphisms from both the blocks in regulating sMBL levels and RVVI risk.

Thus, permutation analysis of haplotype with phenotype suggested that variants of two haplotype blocks have more of a combined effect than the independent effect in regulating sMBL levels and hence RVVI risk. These results are in consonance with the studies suggesting that there are additional 5′ variants as well as SNPs in 3′ haplotype block along with secretor polymorphisms modifies MBL function and its circulating levels and further increases risk of diseases [31, 41–43]. However, further functional studies are needed to confirm these observations and the proposed functional mechanism of these polymorphisms observed by in silico analyses towards disease development. Other than these findings, some other significant associations also observed in the present study that was unexplainable due to unexpected pattern of the variants, suggesting the possibility of other additional variants falling outside the region of observed haplotype blocks.

Our previous investigation suggested the effect of rs3901533 *CLEC7A* SNP in modulating sMBL levels, suggesting RVVI, a polygenic phenotype. Therefore, the interactions between genes i.e. *MBL2* and *CLEC7A* were assessed using multifactor dimensionality reduction (MDR) analysis. Three gene–gene interaction models involving uni-variant, bi-variant and tri-variant appeared as significant predictors of RVVI risk. Of which, the one-way interaction model including *MBL2* Y/X polymorphism with maximum cross-validation consistency (CVC) of 10/10 was found to be the best model for susceptibility to RVVI for pooled RVVI patients and controls. This strongly suggested *MBL2* Y/X polymorphism as the fundamental candidate genetic variant that determining RVVI risk. In addition, two-way interaction model (*MBL2*.rs36014597 and *MBL2*.Y/X) and three-way interaction model (*MBL2*.Y/X, *MBL2*.rs10824792 and *CLEC7A*.rs3901533) also showed association with RVVI risk, though with comparatively low CV consistency to uni-variant model. This further suggests the contribution of both *MBL2* (5′ and 3′ block) and *CLEC7A* genes in the pathogenesis of the RVVI. As per our finest knowledge, this is the first interaction study that found significant associations of genetic interaction in susceptibility to RVVI. However, in the rule as regards with CVC, the three loci model was not the best model for vulnerability to RVVI. The dendrograms and interaction entropy models also suggested a weak synergistic interaction between the variants of three loci model in predicting RVVI risk. In consonance with these findings, our previous results did not show any significant correlation pattern between sMBL and sDectin-1 levels in cases and controls [12]. This further implies, an independent effect of MBL and Dectin-1 to counter the same antigenic stimuli i.e. RVVI. However, analyses of additional putative functional variants especially in *CLEC7A* may reveal a synergy between the two molecules. A single study has shown that opsonophagocytosis of pathogens mediated by MBL leads to an increased intracellular expression of Dectin-1 [44]. This study suggested the inhibitory effect on the phagocytosis of *C. albicans* by neutrophils owing to the Dectin-1 blockage was completely compensated by the exogenous MBL, attributed to its direct role in opsonophagocytosis of pathogens, which is further supported by several receptors e.g. calreticulin and complement receptor 1 present on phagocytes surface that bind to MBL and mediate uptake and phagocytosis of MBL-pathogen complex, independent of complement activation. This further indicates that in the case of infections by *C. albicans*, the interplay between the MBL and Dectin-1 would have a compensatory characteristic, which in turn change the view of synergism mechanism of these molecules in specific situations.

Further, estimation of serum MBL levels was preferred in the present study than methods like mRNA expression. This is because the gene mRNA expression profile does not exactly reflect its phenotypic level in the serum. This difference may possibly be due to protein translational failure or failure of higher order proteins due to variant monomers formation. The same was depicted by a recent study that found low sMBL levels in RVVC cases with high MBL mRNA expression [10]. In addition, proteins with variant allele are suggested to be functionally inactive and are more likely to degrade relative to the proteins with no variations [45]. The functional implication of these variations on phagocytosis or complement activation by MBL warrants further investigations.

## Conclusions

The study presented a low-cost reproducible screening design for additional 5′ variants i.e. rs11003124, rs7084554, rs36014597 and rs11003123 of *MBL2* that can act as markers of susceptibility for vulvovaginal infections or any other diseases. Evaluation of these variants revealed two SNPs i.e. rs7084554 and rs36014597 that are significantly predisposing individuals to RVVI and its types by altering sMBL levels. The LD analyses of the SNP map of *MBL2* indicated two haplotype blocks inside the gene. Permutation analysis of haplotype with phenotype suggested that variants of two blocks have more of a combined effect than the independent effect in regulating sMBL levels and hence RVVI risk, in which associated variants from both the blocks played the crucial role. In consonance with literature, sMBL levels did not exactly associate with standard secretor haplotypes, signifying the role of additional regulating variants of *MBL2*, which possibly be altering sMBL levels. Thus, inclusion of additional regulating variants of *MBL2* in the present study has helped to solve these inconsistencies. The study presented weak synergistic interaction between *MBL2* and *CLEC7A* in association with RVVI risk. However, analyses of additional putative functional variants especially in *CLEC7A* may possibly have simplified the nature of interactions among the polymorphisms and genes studied and might have provided a better understanding of the pathway implicated in the pathogenesis of RVVI. All these preliminary findings of the present study, demand further in-depth functional investigations to clarify the connection observed within and between genes in susceptibility to RVVI. Such kind of studies with larger data-sets will provide invaluable data to authenticate the therapeutic possibilities of MBL for RVVI and its types. Once validated, the day is not so far when different MBL formulations will be available over the counter for the RVVI treatment. Because, MBL replacement therapy has already passed the phase I clinical trials to aid patients with MBL paucity [46–49]. While, recombinant MBL production, Phase II and phase III trials are presently in progress. However, in contrast to MBL, more extensive research is needed to dissect the complete and specific role of Dectin-1 in RVVI, prior to judging its therapeutic potential.

## Additional files

**Additional file 1.** Distribution and comparison of 5′ block haplotypes of *MBL2* in cases and controls.

**Additional file 2.** Distribution and comparison of combined 5′/3′ haplotypes of *MBL2* in cases and controls.

**Additional file 3.** *MBL2* SNPs frequencies in present study population (North Indian) and modern human populations in human genomes variation project i.e. The 1000 Genomes Project (Phase 3).

## Data Availability

The data that support the findings of this study are available from the corresponding author upon reasonable request.
