# Supplementary material for: Impact of SNPs interplay across the locus of MBL2, between MBL and Dectin-1 gene, on women’s risk of developing recurrent vulvovaginal infections
Source: Cell Biosci. 2019 May 7;9:35. doi: 10.1186/s13578-019-0300-4 (PMC6505208; doi:10.1186/s13578-019-0300-4)
Supplement: Supplementary file 1 — Additional file 1. Distribution and comparison of 5′ block haplotypes of MBL2 in cases and controls. [file 13578_2019_300_MOESM1_ESM.docx]

**Additional file 1**: Distribution and comparison of 5´ block haplotypes of *MBL2* in cases and controls.

| **Haplotypes**  **(5´🡪3´)** | **No. (%) of Controls** | **No. (%) of Cases** | | | | **Haplotype Comparison** | | | | | | | |
| --- | --- | --- | --- | --- | --- | --- | --- | --- | --- | --- | --- | --- | --- |
|  |  |  |  |  |  |  | | | | | | | |
|  |  | **Total RVVI**  **Cases** | **Types of RVVI** | | | **RVVI *vs* Controls** | | **BV *vs* Controls** | | **VVC *vs* Controls** | | **MI *vs* Controls** | |
|  | **(n=406)** | **(n=516)** | **BV**  **(n=194)** | **VVC (n=124)** | **MI**  **(n=82)** | **OR**  **(95% CI)** | **p-value** | **OR**  **(95% CI)** | **p-value** | **OR**  **(95% CI)** | **p-value** | **OR**  **(95%CI)** | **p-value** |
| **Common** | | | | | | | | | | | | | |
| HTTAXGQB (5′H-1) | 52 (12.80) | 98 (18.99) | 33 (17.01) | 20 (16.12) | 16 (19.51) | 1 |  | 1 |  | 1 |  | 1 |  |
| HTTAYGQB (5′H-2) | 57 (14.03) | 50 (9.68) | 19 (9.79) | 16 (12.90) | 6 (7.31) | 0.46  (0.28 - 0.77) | 0.003****** | 0.52  (0.26 - 1.03) | 0.062 | 0.72  (0.34 - 1.55) | 0.415 | 0.34  (0.12 - 0.94) | 0.03****** |
| LTTAXGQB (5′H-3) | 39 (9.60) | 65 (12.59) | 27 (13.91) | 17 (13.70) | 7 (8.53) | 0.88  (0.52 - 1.48) | 0.643 | 1.09  (0.56 - 2.10) | 0.795 | 1.13  (0.52 - 2.44) | 0.749 | 0.58  (0.21 - 1.55) | 0.281 |
| LTTAYGPA (5′H-4) | 51 (12.56) | 51 (9.88) | 12 (6.18) | 14 (11.29) | 10 (12.19) | 0.53  (0.31 - 0.88) | 0.01****** | 0.37  (0.17 - 0.79) | 0.01****** | 0.71  (0.32 - 1.56) | 0.399 | 0.63  (0.26 - 1.53) | 0.315 |
| LGCGYAPA (5′H-5) | 24 (5.91) | 64 (12.40) | 19 (9.79) | 14 (11.29) | 14 (17.07) | 1.41  (0.79 - 2.52) | 0.238 | 0.55  (0.28 - 1.09) | 0.089 | 1.51  (0.65 - 3.50) | 0.329 | 1.89  (0.79 - 4.50) | 0.147 |
| HTTAYAPA (5′H-6) | 17 (4.18) | 26 (5.03) | 11 (5.67) | 6 (4.83) | 3 (3.65) | 0.81  (0.40 - 1.63) | 0.557 | 1.01  (0.42 - 2.44) | 0.965 | 0.91  (0.31 - 2.65) | 0.874 | 0.57  (0.14 - 2.21) | 0.419 |
| **Rare** | | | | | | | | | | | | | |
| LTTAYAPA (5′H-7) | 18 (4.43) | 7 (1.35) | 3 (1.54) | 3 (2.41) | 0 (0.00) | 0.20  (0.08 - 0.52) | 0.0009******* | 0.26  (0.07 - 0.96) | 0.043***** | 0.43  (0.11 - 1.63) | 0.216 | - | NA |
| LTTAXGPA (5′H-8) | 11 (2.70) | 13 (2.51) | 5 (2.57) | 2 (1.61) | 5 (6.09) | 0.62  (0.26 - 1.49) | 0.293 | 0.71  (0.22 - 2.24) | 0.567 | 0.47  (0.09 - 2.32) | 0.356 | 1.47  (0.44 - 4.88) | 0.522 |
| HTTAYGPA (5′H-9) | 13 (3.20) | 9 (1.74) | 7 (3.60) | 2 (1.61) | 0 (0.00) | 0.36  (0.14 - 0.91) | 0.031***** | 0.84  (0.30 - 2.34) | 0.751 | 0.40  (0.08 - 1.93) | 0.254 | - | NA |
| LTTAYGQB (5′H-10) | 14 (3.44) | 7 (1.35) | 1 (0.51) | 3 (2.41) | 2 (2.43) | 0.26  (0.10 - 0.69) | 0.007****** | 0.11  (0.01 - 0.89) | 0.039***** | 0.55  (0.14 - 2.14) | 0.395 | 0.46  (0.09 - 2.26) | 0.342 |
| LGTGYAPA (5′H-11) | 12 (2.95) | 7 (1.35) | 3 (1.54) | 1 (0.80) | 1 (1.21) | 0.30  (0.11 - 0.83) | 0.02***** | 0.39  (0.10 - 1.50) | 0.172 | 0.21  (0.02 - 1.77) | 0.154 | 0.27  (0.03 - 2.24) | 0.226 |
| LTCGYAPA (5′H-12) | 4 (0.98) | 13 (2.51) | 8 (4.12) | 3 (2.41) | 1 (1.21) | 1.72  (0.53 - 5.55) | 0.361 | 3.15  (0.87 - 11.30) | 0.078 | 1.95  (0.40 - 9.49) | 0.408 | 0.81  (0.08 - 7.80) | 0.857 |
| LGTAYAPA (5′H-13) | 15 (3.69) | 2 (0.38) | 0 (0.00) | 1 (0.80) | 0 (0.00) | 0.07  (0.01 - 0.32) | 0.0006******* | - | NA | 0.17  (0.02 - 1.39) | 0.100 | - | NA |
| LTTGYAPA (5′H-14) | 4 (0.98) | 10 (1.93) | 5 (2.57) | 1 (0.80) | 1 (1.21) | 1.32  (0.39 - 4.43) | 0.646 | 1.96  (0.49 - 7.87) | 0.337 | 0.65  (0.06 - 6.17) | 0.707 | 0.81  (0.08 - 7.80) | 0.857 |
| LTCAYGPA (5′H-15) | 9 (2.21) | 5 (0.96) | 4 (2.06) | 1 (0.80) | 0 (0.00) | 0.29  (0.09 - 0.92) | 0.036***** | 0.70  (0.19 - 2.45) | 0.578 | 0.28  (0.03 - 2.42) | 0.253 | - | NA |
| HTTGYGPA (5′H-16) | 2 (0.49) | 11 (2.13) | 7 (3.60) | 1 (0.80) | 0 (0.00) | 2.91  (0.62 - 13.66) | 0.173 | 5.51  (1.07 - 28.17) | 0.04***** | 1.30  (0.11 - 15.14) | 0.834 | - | NA |
| HGCAYAQB (5′H-17) | 8 (1.97) | 3 (0.58) | 2 (1.03) | 1 (0.80) | 0 (0.00) | 0.19  (0.05 - 0.78) | 0.02***** | 0.39  (0.07 - 1.97) | 0.256 | 0.32  (0.03 - 2.76) | 0.303 | - | NA |
| HGCGYAQB (5′H-18) | 7 (1.72) | 4 (0.77) | 1 (0.51) | 0 (0.00) | 1 (1.21) | 0.30  (0.08 - 1.08) | 0.066 | 0.22  (0.02 - 1.91) | 0.172 | - | NA | 0.46  (0.05 - 4.06) | 0.488 |
| LGCGXAPA (5′H-19) | 1 (0.24) | 9 (1.74) | 2 (1.03) | 3 (2.41) | 1 (1.21) | 4.77  (0.58 - 38.73) | 0.143 | 3.15  (0.27 - 36.15) | 0.356 | 7.80  (0.76 - 79.46) | 0.082 | 3.25  (0.19 - 54.95) | 0.414 |
| LGCGYGPA (5′H-20) | 2 (0.49) | 5 (0.96) | 0 (0.00) | 2 (1.61) | 0 (0.00) | 1.32  (0.24 - 7.07) | 0.740 | - | NA | 2.60  (0.34 - 19.73) | 0.355 | - | NA |
| LTTGYGPA (5′H-21) | 1 (0.24) | 5 (0.96) | 1 (0.51) | 3 (2.41) | 0 (0.00) | 2.65  (0.30 - 23.31) | 0.378 | 1.57  (0.09 - 26.06) | 0.750 | 7.80  (0.76 - 79.46) | 0.082 | - | NA |
| LGCAYAQB (5′H-22) | 6 (1.47) | 0 (0.00) | 0 (0.00) | 0 (0.00) | 0 (0.00) | - | NA | - | NA | - | NA | - | NA |
| LTTAXAPA (5′H-23) | 4 (0.98) | 1 (0.19) | 0 (0.00) | 1 (0.80) | 0 (0.00) | 0.13  (0.01 - 1.21) | 0.074 | - | NA | 0.65  (0.06 - 6.17) | 0.707 | - | NA |
| LTTAYGQA (5′H-24) | 3 (0.73) | 1 (0.19) | 1 (0.51) | 0 (0.00) | 0 (0.00) | 0.17  (0.01 - 1.74) | 0.137 | 0.52  (0.05 - 5.26) | 0.584 | - | NA | - | NA |
| LTTGXGPA (5′H-25) | 1 (0.24) | 3 (0.58) | 1 (0.51) | 1 (0.80) | 1 (1.21) | 1.59  (0.16 - 15.68) | 0.690 | 1.57  (0.09 - 26.0) | 0.750 | 2.60  (0.15 - 43.59) | 0.506 | 3.25  (0.19 - 54.95) | 0.414 |
| LGCGYAQB (5′H-26) | 2 (0.49) | 2 (0.38) | 1 (0.51) | 0 (0.00) | 1 (1.21) | 0.53  (0.07 - 3.87) | 0.532 | 0.78  (0.06 - 9.03) | 0.848 | - | NA | 1.62  (0.13 - 19.11) | 0.699 |
| HTTAYAQB (5′H-27) | 4 (0.98) | 0 (0.00) | 0 (0.00) | 0 (0.00) | 0 (0.00) | - | NA | - | NA | - | NA | - | NA |
| HTTGYAPA (5′H-28) | 1 (0.24) | 3 (0.58) | 2 (1.03) | 0 (0.00) | 0 (0.00) | 1.59  (0.16 - 15.68) | 0.690 | 3.15  (0.27 - 36.15) | 0.356 | - | NA | - | NA |
| HTCGXAQB (5′H-29) | 0 (0.00) | 4 (0.77) | 2 (1.03) | 0 (0.00) | 1 (1.21) | - | NA | - | NA | - | NA | - | NA |
| HGTAYGQB (5′H-30) | 3 (0.73) | 1 (0.19) | 1 (0.51) | 0 (0.00) | 0 (0.00) | 0.17  (0.01 - 1.74) | 0.137 | 0.52  (0.05 - 5.26) | 0.584 | - | NA | - | NA |
| HGCGXAQB (5′H-31) | 0 (0.00) | 4 (0.77) | 3 (1.54) | 0 (0.00) | 1 (1.21) | - | NA | - | NA | - | NA | - | NA |
| LGCGXAQB (5′H-32) | 0 (0.00) | 4 (0.77) | 3 (1.54) | 0 (0.00) | 1 (1.21) | - | NA | - | NA | - | NA | - | NA |
| LTTGYGQB (5′H-33) | 0 (0.00) | 3 (0.58) | 0 (0.00) | 2 (1.61) | 1 (1.21) | - | NA | - | NA | - | NA | - | NA |
| LGTAYGPA (5′H-34) | 2 (0.49) | 1 (0.19) | 0 (0.00) | 1 (0.80) | 0 (0.00) | 0.26  (0.02 - 2.99) | 0.283 | - | NA | 1.30  (0.11 - 15.14) | 0.834 | - | NA |
| HTTAXGPA (5′H-35) | 0 (0.00) | 3 (0.58) | 2 (1.03) | 0 (0.00) | 1 (1.21) | - | NA | - | NA | - | NA | - | NA |
| HTTAXGPB (5′H-36) | 0 (0.00) | 3 (0.58) | 0 (0.00) | 1 (0.80) | 2 (2.43) | - | NA | - | NA | - | NA | - | NA |
| HTTAXAQB (5′H-37) | 2 (0.49) | 1 (0.19) | 1 (0.51) | 0 (0.00) | 0 (0.00) | 0.26  (0.02 - 2.99) | 0.283 | 0.78  (0.06 - 9.03) | 0.848 | - | NA | - | NA |
| HGCGYAPA (5′H-38) | 0 (0.00) | 3 (0.58) | 1 (0.51) | 1 (0.80) | 1 (1.21) | - | NA | - | NA | - | NA | - | NA |
| LTTAXAQB (5′H-39) | 1 (0.24) | 1 (0.19) | 1 (0.51) | 0 (0.00) | 0 (0.00) | 0.53  (0.03 - 8.65) | 0.656 | 1.57  (0.09 - 26.06) | 0.750 | - | NA | - | NA |
| LTTAYAQB (5′H-40) | 2 (0.49) | 0 (0.00) | 0 (0.00) | 0 (0.00) | 0 (0.00) | - | NA | - | NA | - | NA | - | NA |
| LTCGYGPA (5′H-41) | 1 (0.24) | 1 (0.19) | 1 (0.51) | 0 (0.00) | 0 (0.00) | 0.53  (0.03 - 8.65) | 0.656 | 1.57  (0.09 - 26.06) | 0.750 | - | NA | - | NA |
| LGTAXGQB (5′H-42) | 0 (0.00) | 2 (0.38) | 1 (0.51) | 1 (0.80) | 0 (0.00) | - | NA | - | NA | - | NA | - | NA |
| LGTGYGPA (5′H-43) | 1 (0.24) | 1 (0.19) | 1 (0.51) | 0 (0.00) | 0 (0.00) | 0.53  (0.03 - 8.65) | 0.656 | 1.57  (0.09 - 26.06) | 0.750 | - | NA | - | NA |
| LGCAXAQB (5′H-44) | 0 (0.00) | 2 (0.38) | 0 (0.00) | 1 (0.80) | 0 (0.00) | - | NA | - | NA | - | NA | - | NA |
| LGCAYGPA (5′H-45) | 2 (0.49) | 0 (0.00) | 0 (0.00) | 0 (0.00) | 0 (0.00) | - | NA | - | NA | - | NA | - | NA |
| LGCGXGQB (5′H-46) | 2 (0.49) | 0 (0.00) | 0 (0.00) | 0 (0.00) | 0 (0.00) | - | NA | - | NA | - | NA | - | NA |
| HTTAXAPA (5′H-47) | 1 (0.24) | 1 (0.19) | 0 (0.00) | 0 (0.00) | 1 (1.21) | 0.53  (0.03 - 8.65) | 0.656 | - | NA | - | NA |  |  |
| HTTAYGPB (5′H-48) | 0 (0.00) | 2 (0.38) | 0 (0.00) | 0 (0.00) | 2 (2.43) | - | NA | - | NA | - | NA | - | NA |
| HGCGYGQB (5′H-49) | 1 (0.24) | 1 (0.19) | 0 (0.00) | 0 (0.00) | 0 (0.00) | 0.53  (0.03 - 8.65) | 0.656 | - | NA | - | NA | - | NA |
| LTTAYGPB (5′H-50) | 1 (0.24) | 0 (0.00) | 0 (0.00) | 0 (0.00) | 0 (0.00) | - | NA | - | NA | - | NA | - | NA |
| LTCAXGPA (5′H-51) | 1 (0.24) | 0 (0.00) | 0 (0.00) | 0 (0.00) | 0 (0.00) | - | NA | - | NA | - | NA | - | NA |
| LGTGYAQB (5′H-52) | 1 (0.24) | 0 (0.00) | 0 (0.00) | 0 (0.00) | 0 (0.00) | - | NA | - | NA | - | NA | - | NA |
| LGCAYGQB (5′H-53) | 1 (0.24) | 0 (0.00) | 0 (0.00) | 0 (0.00) | 0 (0.00) | - | NA | - | NA | - | NA | - | NA |
| HTTAXGQA (5′H-54) | 0 (0.00) | 1 (0.19) | 1 (0.51) | 0 (0.00) | 0 (0.00) | - | NA | - | NA | - | NA | - | NA |
| HTCGYGPA (5′H-55) | 0 (0.00) | 1 (0.19) | 0 (0.00) | 1 (0.80) | 0 (0.00) | - | NA | - | NA | - | NA | - | NA |
| HTCGYAPA (5′H-56) | 0 (0.00) | 1 (0.19) | 0 (0.00) | 0 (0.00) | 1 (1.21) | - | NA | - | NA | - | NA | - | NA |
| HGTAXGQB (5′H-57) | 1 (0.24) | 0 (0.00) | 0 (0.00) | 0 (0.00) | 0 (0.00) | - | NA | - | NA | - | NA | - | NA |
| HGTGYGPA (5′H-58) | 1 (0.24) | 0 (0.00) | 0 (0.00) | 0 (0.00) | 0 (0.00) | - | NA | - | NA | - | NA | - | NA |
| HGCGXAPA (5′H-59) | 0 (0.00) | 1 (0.19) | 1 (0.51) | 0 (0.00) | 0 (0.00) | - | NA | - | NA | - | NA | - | NA |
| Global p-value for case/control haplotype association was 0.01,; NA, not applicable.*****p≤0.05; ******p≤0.01; *******p≤0.001. | | | | | | | | | | | | | |
