# Supplementary material for: Impact of SNPs interplay across the locus of MBL2, between MBL and Dectin-1 gene, on women’s risk of developing recurrent vulvovaginal infections
Source: Cell Biosci. 2019 May 7;9:35. doi: 10.1186/s13578-019-0300-4 (PMC6505208; doi:10.1186/s13578-019-0300-4)
Supplement: Supplementary file 2 — Additional file 2. Distribution and comparison of combined 5′/3′ haplotypes of MBL2 in cases and controls. [file 13578_2019_300_MOESM2_ESM.docx]

**Additional file 2:** Distribution and comparison of combined 5′/3′ haplotypes of *MBL2* in cases and controls.

| **Haplotypes**  **(5´🡪3´)** | **No. (%) of Controls** | **No. (%) of Cases** | | | | **Haplotype Comparison** | | | | | | | |
| --- | --- | --- | --- | --- | --- | --- | --- | --- | --- | --- | --- | --- | --- |
|  |  |  |  |  |  |  | | | | | | | |
|  |  | **Total RVVI**  **Cases** | **Types of RVVI** | | | **RVVI *vs* Controls** | | **BV *vs* Controls** | | **VVC *vs* Controls** | | **MI *vs* Controls** | |
|  | **(n=218)** | **(n=218)** | **BV (n=112)** | **VVC (n=56)** | **MI**  **(n=50)** | **OR**  **(95% CI)** | **p-value** | **OR**  **(95% CI)** | **p-value** | **OR**  **(95% CI)** | **p-value** | **OR**  **(95%CI)** | **p-value** |
| **Common** | | | | | | | | | | | | | |
| 5′H-10/3′H-2 | 24(11.00) | 23 (10.55) | 9 (8.03) | 8 (14.2) | 6 (12.00) | 1 |  | 1 |  | 1 |  | 1 |  |
| 5′H-5/3′H-1 | 12 (5.50) | 16 (7.33) | 9 (8.03) | 3 (5.35) | 4 (8.00) | 1.39  (0.54 - 3.56) | 0.492 | 2.00  (0.63 - 6.34) | 0.2395 | 0.75  (0.16 - 3.35) | 0.706 | 1.33  (0.31 - 5.64) | 0.695 |
| 5′H-3/3′H-3 | 15 (6.88) | 10 (4.58) | 4 (3.57) | 3 (5.35) | 3 (6.00) | 0.69  (0.26 - 1.86) | 0.469 | 0.71  (0.18 - 2.72) | 0.618 | 0.60  (0.13 - 2.62) | 0.497 | 0.80  (0.17 - 3.68) | 0.774 |
| 5′H-1/3′H-1 | 14 (6.42) | 10 (4.58) | 10 (8.92) | 0 (0.00) | 0 (0.00) | 0.74  (0.27 - 2.01) | 0.561 | 1.90  (0.62 - 5.81) | 0.257 | - | NA | - | NA |
| 5′H-9/3′H-1 | 15 (6.88) | 8 (3.66) | 4 (3.57) | 2 (3.57) | 2 (4.00) | 0.55  (0.19 - 1.56) | 0.265 | 0.71  (0.18 - 2.72) | 0.618 | 0.40  (0.07 - 2.14) | 0.284 | 0.53  (0.09 - 2.99) | 0.475 |
| 5′H-1/3′H-2 | 9 (4.12) | 11 (5.04) | 4 (3.57) | 5 (8.92 | 2 (4.00) | 1.27  (0.44 - 3.64) | 0.649 | 1.18  (0.29 - 4.83) | 0.812 | 1.66  (0.43 - 6.45) | 0.459 | 0.88  (0.15 - 5.24) | 0.896 |
| 5′H-35/3′H-3 | 4 (1.83) | 11 (5.04) | 5 (4.46) | 3 (5.35) | 3 (6.00) | 2.86  (0.79 - 10.31) | 0.106 | 3.33  (0.72 - 15.26) | 0.121 | 2.25  (0.41 - 12.28) | 0.349 | 3.00  (0.52 - 17.15) | 0.216 |
| 5′H-60/3′H-1 | 3 (1.37) | 11 (5.04) | 7 (6.25) | 3 (5.35) | 1 (2.00) | 3.82  (0.94 - 15.49) | 0.060 | 6.22  (1.31 - 29.44) | 0.021***** | 3.00  (0.50 - 17.95) | 0.228 | 1.33  (0.11 - 15.20) | 0.816 |
| **Rare** | | | | | | | | | | | | | |
| 5′H-6/3′H-1 | 9 (4.12) | 8 (3.66) | 4 (3.57) | 1 (1.78) | 3 (6.00) | 0.92  (0.30 - 2.81) | 0.894 | 1.18  (0.29 - 4.83) | 0.812 | 0.33  (0.03 - 3.05) | 0.331 | 1.33  (0.27 - 6.49) | 0.721 |
| 5′H-60/3′H-3 | 7 (3.21) | 9 (4.12) | 5 (4.46) | 3 (5.35) | 1 (2.00) | 1.34  (0.42 - 4.20) | 0.613 | 1.90  (0.47 - 7.57) | 0.360 | 1.28  (0.26 - 6.18) | 0.753 | 0.57  (0.05 - 5.57) | 0.630 |
| 5′H-4/3′H-1 | 10 (4.58) | 3 (1.37) | 2 (1.78) | 0 (0.00) | 1 (2.00) | 0.31  (0.07 - 1.28) | 0.106 | 0.53  (0.09 - 2.92) | 0.468 | - | NA | 0.40  (0.042 - 3.76) | 0.423 |
| 5′H-19/3′H-1 | 2 (0.91) | 9 (4.12) | 3 (2.67) | 3 (5.35) | 3 (6.00) | 4.69  (0.91 - 24.09) | 0.063 | 4.00  (0.57 - 28.01) | 0.162 | 4.50  (0.63 - 31.94) | 0.132 | 6.00  (0.81 - 44.35) | 0.079 |
| 5′H-3/3′H-1 | 6 (2.75) | 3 (1.37) | 2 (1.78) | 0 (0.00) | 1 (2.00) | 0.52  (0.11 - 2.33) | 0.395 | 0.88  (0.15 - 5.24) | 0.896 | - | NA | 0.66  (0.067 - 6.63) | 0.729 |
| 5′H-3/3′H-2 | 1 (0.45) | 7 (3.21) | 4 (3.57) | 1 (1.78) | 2 (4.00) | 7.30  (0.83 - 64.10) | 0.072 | 10.66  (1.04 - 108.69) | 0.045***** | 3.00  (0.16 - 53.71) | 0.455 | 8.00  (0.61 - 103.67) | 0.111 |
| 5′H-35/3′H-1 | 4 (1.83) | 4 (1.83) | 4 (3.57) | 0 (0.00) | 0 (0.00) | 1.04  (0.23 - 4.67) | 0.955 | 2.66  (0.54 - 12.99) | 0.224 | - | NA | - | NA |
| 5′H-8/3′H-1 | 4 (1.83) | 4 (1.83) | 2 (1.78) | 1 (1.78) | 1 (2.00) | 1.04  (0.23 - 4.67) | 0.955 | 1.33  (0.20 - 8.58) | 0.762 | 0.75  (0.07 - 7.73) | 0.809 | 1.00  (0.09 - 10.66) | 1.00 |
| 5′H-16/3′H-1 | 4 (1.83) | 2 (0.91) | 1 (0.89) | 1 (1.78) | 0 (0.00) | 0.52  (0.08 - 3.12) | 0.476 | 0.66  (0.06 - 6.79) | 0.732 | 0.75  (0.07 - 7.73) | 0.809 | - | NA |
| 5′H-18/3′H-1 | 3 (1.37) | 3 (1.37) | 1 (0.89) | 0 (0.00) | 2 (4.00) | 1.04  (0.19 - 5.70) | 0.960 | 0.88  (0.08 - 9.69) | 0.923 | - | NA | 2.66  (0.36 - 19.71) | 0.33 |
| 5′H-4/3′H-3 | 3 (1.37) | 3 (1.37) | 0 (0.00) | 2 (3.57) | 1 (2.00) | 1.04  (0.19 - 5.70) | 0.960 | - | NA | 2.00  (0.28 - 14.19) | 0.488 | 1.33  (0.11 - 15.20) | 0.816 |
| 5′H-40/3′H-2 | 4 (1.83) | 2 (0.91) | 2 (1.78) | 0 (0.00) | 0 (0.00) | 0.52  (0.08 - 3.12) | 0.4765 | 1.33  (0.20 - 8.58) | 0.762 | - | NA | - | NA |
| 5′H-8/3′H-3 | 2 (0.91) | 4 (1.83) | 3 (2.67) | 0 (0.00) | 1 (2.00) | 2.08  (0.34 - 12.51) | 0.420 | 4.00  (0.57 - 28.01) | 0.162 | - | NA | 2.00  (0.15 - 25.91) | 0.595 |
| 5′H-11/3′H-1 | 4 (1.83) | 2 (0.91) | 0 (0.00) | 1 (1.78) | 1 (2.00) | 0.52  (0.08 - 3.12) | 0.476 | - | NA | 0.75  (0.07 - 7.73) | 0.809 | 1.00  (0.09 - 10.66) | 1.000 |
| 5′H-61/3′H-1 | 5 (2.29) | 0 (0.00) | 0 (0.00) | 0 (0.00) | 0 (0.00) | - | NA | - | NA | - | NA | - | NA |
| 5′H-12/3′H-1 | 2 (0.91) | 3 (1.37) | 2 (1.78) | 1 (1.78) | 0 (0.00) | 1.56  (0.23 - 10.24) | 0.640 | 2.66  (0.32 - 21.87) | 0.361 | 1.50  (0.11 - 18.83) | 0.753 | - | NA |
| 5′H-13/3′H-1 | 4 (1.83) | 1 (0.45) | 0 (0.00) | 1 (1.78) | 0 (0.00) | 0.26  (0.02 - 2.51) | 0.244 | - | NA | 0.75  (0.07 - 7.73) | 0.809 | - | NA |
| 5′H-10/3′H-3 | 2 (0.91) | 2 (0.91) | 0 (0.00) | 1 (1.78) | 1 (2.00) | 1.04  (0.13 - 8.03) | 0.967 | - | NA | 1.50  (0.11 - 18.83) | 0.753 | 2.00  (0.15 - 25.91) | 0.595 |
| 5′H-23/3′H-1 | 3 (1.37) | 1 (0.45) | 0 (0.00) | 1 (1.78) | 0 (0.00) | 0.34  (0.03 - 3.59) | 0.375 | - | NA | 1.00  (0.09 - 11.02) | 1.000 | - | NA |
| 5′H-62/3′H-3 | 2 (0.91) | 2 (0.91) | 0 (0.00) | 1 (1.78) | 1 (2.00) | 1.04  (0.13 - 8.03) | 0.967 | - | NA | 1.50  (0.11 - 18.83) | 0.753 | 2.00  (0.15 - 25.91) | 0.595 |
| 5′H-34/3′H-1 | 3 (1.37) | 1 (0.45) | 0 (0.00) | 1 (1.78) | 0 (0.00) | 0.34  (0.03 - 3.59) | 0.375 | - | NA | 1.00  (0.09 - 11.02) | 1.000 | - | NA |
| 5′H-28/3′H-1 | 0 (0.00) | 3 (1.37) | 3 (2.67) | 0 (0.00) | 0 (0.00) | - | NA | - | NA | - | NA | - | NA |
| 5′H-63/3′H-1 | 3 (1.37) | 0 (0.00) | 0 (0.00) | 0 (0.00) | 0 (0.00) | - | NA | - | NA | - | NA | - | NA |
| 5′H-38/3′H-1 | 2 (0.91) | 1 (0.45) | 1 (0.89) | 0 (0.00) | 0 (0.00) | 0.52  (0.04 - 6.15) | 0.605 | 1.33  (0.10 - 16.56) | 0.822 | - | NA | - | NA |
| 5′H-8/3′H-2 | 0 (0.00) | 3 (1.37) | 1 (0.89) | 1 (1.78) | 1 (2.00) | - | NA | - | NA | - | NA | - | NA |
| 5′H-15/3′H-1 | 2 (0.91) | 1 (0.45) | 0 (0.00) | 1 (1.78) | 0 (0.00) | 0.52  (0.04 - 6.15) | 0.605 | - | NA | 1.50  (0.11 - 18.83) | 0.753 | - | NA |
| 5′H-64/3′H-1 | 3 (1.37) | 0 (0.00) | 0 (0.00) | 0 (0.00) | 0 (0.00) | - | NA | - | NA | - | NA | - | NA |
| 5′H-5/3′H-3 | 0 (0.00) | 3 (1.37) | 0 (0.00) | 2 (3.57) | 1 (2.00) | - | NA | - | NA | - | NA | - | NA |
| 5′H-9/3′H-3 | 1 (0.45) | 1 (0.45) | 0 (0.00) | 0 (0.00) | 1 (2.00) | 1.04  (0.06 - 17.68) | 0.976 | - | NA | - | NA | 4.00  (0.21 - 73.62) | 0.350 |
| 5′H-48/3′H-1 | 0 (0.00) | 2 (0.91) | 0 (0.00) | 0 (0.00) | 2 (4.00) | - | NA | - | NA | - | NA | - | NA |
| 5′H-36/3′H-1 | 0 (0.00) | 2 (0.91) | 0 (0.00) | 0 (0.00) | 2 (4.00) | - | NA | - | NA | - | NA | - | NA |
| 5′H-37/3′H-2 | 1 (0.45) | 1 (0.45) | 0 (0.00) | 0 (0.00) | 1 (2.00) | 1.04  (0.06 - 17.68) | 0.976 | - | NA | - | NA | 4.00  (0.21 - 73.62) | 0.350 |
| 5′H-65/3′H-2 | 0 (0.00) | 2 (0.91) | 1 (0.89) | 0 (0.00) | 1 (2.00) | - | NA | - | NA | - | NA | - | NA |
| 5′H-24/3′H-1 | 2 (0.91) | 0 (0.00) | 0 (0.00) | 0 (0.00) | 0 (0.00) | - | NA | - | NA | - | NA | - | NA |
| 5′H-40/3′H-1 | 2 (0.91) | 0 (0.00) | 0 (0.00) | 0 (0.00) | 0 (0.00) | - | NA | - | NA | - | NA | - | NA |
| 5′H-21/3′H-2 | 0 (0.00) | 2 (0.91) | 1 (0.89) | 0 (0.00) | 1 (2.00) | - | NA | - | NA | - | NA | - | NA |
| 5′H-66/3′H-1 | 2 (0.91) | 0 (0.00) | 0 (0.00) | 0 (0.00) | 0 (0.00) | - | NA | - | NA | - | NA | - | NA |
| 5′H-52/3′H-2 | 2 (0.91) | 0 (0.00) | 0 (0.00) | 0 (0.00) | 0 (0.00) | - | NA | - | NA | - | NA | - | NA |
| 5′H-26/3′H-1 | 1 (0.45) | 1 (0.45) | 1 (0.89) | 0 (0.00) | 0 (0.00) | 1.04  (0.06 - 17.68) | 0.976 | 2.66  (0.15 - 47.30) | 0.503 | - | NA | - | NA |
| 5′H-26/3′H-2 | 1 (0.45) | 1 (0.45) | 1 (0.89) | 0 (0.00) | 0 (0.00) | 1.04  (0.06 - 17.68) | 0.976 | 2.66  (0.15 - 47.30) | 0.503 | - | NA | - | NA |
| 5′H-9/3′H-2 | 1 (0.45) | 0 (0.00) | 0 (0.00) | 0 (0.00) | 0 (0.00) | - | NA | - | NA | - | NA | - | NA |
| 5′H-1/3′H-3 | 1 (0.45) | 0 (0.00) | 0 (0.00) | 0 (0.00) | 0 (0.00) | - | NA | - | NA | - | NA | - | NA |
| 5′H-6/3′H-4 | 0 (0.00) | 1 (0.45) | 1 (0.89) | 0 (0.00) | 0 (0.00) | - | NA | - | NA | - | NA | - | NA |
| 5′H-6/3′H-3 | 1 (0.45) | 0 (0.00) | 0 (0.00) | 0 (0.00) | 0 (0.00) | - | NA | - | NA | - | NA | - | NA |
| 5′H-6/3′H-2 | 0 (0.00) | 1 (0.45) | 1 (0.89) | 0 (0.00) | 0 (0.00) | - | NA | - | NA | - | NA | - | NA |
| 5′H-27/3′H-1 | 0 (0.00) | 1 (0.45) | 0 (0.00) | 1 (1.78) | 0 (0.00) | - | NA | - | NA | - | NA | - | NA |
| 5′H-27/3′H-2 | 1 (0.45) | 0 (0.00) | 0 (0.00) | 0 (0.00) | 0 (0.00) | - | NA | - | NA | - | NA | - | NA |
| 5′H-36/3′H-3 | 0 (0.00) | 1 (0.45) | 0 (0.00) | 1 (1.78) | 0 (0.00) | - | NA | - | NA | - | NA | - | NA |
| 5′H-67/3′H-1 | 0 (0.00) | 1 (0.45) | 1 (0.89) | 0 (0.00) | 0 (0.00) | - | NA | - | NA | - | NA | - | NA |
| 5′H-60/3′H-4 | 0 (0.00) | 1 (0.45) | 1 (0.89) | 0 (0.00) | 0 (0.00) | - | NA | - | NA | - | NA | - | NA |
| 5′H-60/3′H-2 | 1 (0.45) | 0 (0.00) | 0 (0.00) | 0 (0.00) | 0 (0.00) | - | NA | - | NA | - | NA | - | NA |
| 5′H-68/3′H-1 | 0 (0.00) | 1 (0.45) | 1 (0.89) | 0 (0.00) | 0 (0.00) | - | NA | - | NA | - | NA | - | NA |
| 5′H-65/3′H-7 | 0 (0.00) | 1 (0.45) | 0 (0.00) | 1 (1.78) | 0 (0.00) | - | NA | - | NA | - | NA | - | NA |
| 5′H-69/3′H-1 | 0 (0.00) | 1 (0.45) | 1 (0.89) | 0 (0.00) | 0 (0.00) | - | NA | - | NA | - | NA | - | NA |
| 5′H-18/3′H-2 | 1 (0.45) | 0 (0.00) | 0 (0.00) | 0 (0.00) | 0 (0.00) | - | NA | - | NA | - | NA | - | NA |
| 5′H-4/3′H-5 | 1 (0.45) | 0 (0.00) | 0 (0.00) | 0 (0.00) | 0 (0.00) | - | NA | - | NA | - | NA | - | NA |
| 5′H-4/3′H-2 | 1 (0.45) | 0 (0.00) | 0 (0.00) | 0 (0.00) | 0 (0.00) | - | NA | - | NA | - | NA | - | NA |
| 5′H-7/3′H-1 | 0 (0.00) | 1 (0.45) | 1 (0.89) | 0 (0.00) | 0 (0.00) | - | NA | - | NA | - | NA | - | NA |
| 5′H-7/3′H-3 | 0 (0.00) | 1 (0.45) | 0 (0.00) | 1 (1.78) | 0 (0.00) | - | NA | - | NA | - | NA | - | NA |
| 5′H-7/3′H-2 | 1 (0.45) | 0 (0.00) | 0 (0.00) | 0 (0.00) | 0 (0.00) | - | NA | - | NA | - | NA | - | NA |
| 5′H-3/3′H-6 | 0 (0.00) | 1 (0.45) | 0 (0.00) | 1 (1.78) | 0 (0.00) | - | NA | - | NA | - | NA | - | NA |
| 5′H-23/3′H-3 | 1 (0.45) | 0 (0.00) | 0 (0.00) | 0 (0.00) | 0 (0.00) | - | NA | - | NA | - | NA | - | NA |
| 5′H-23/3′H-2 | 0 (0.00) | 1 (0.45) | 1 (0.89) | 0 (0.00) | 0 (0.00) | - | NA | - | NA | - | NA | - | NA |
| 5′H-14/3′H-1 | 0 (0.00) | 1 (0.45) | 1 (0.89) | 0 (0.00) | 0 (0.00) | - | NA | - | NA | - | NA | - | NA |
| 5′H-70/3′H-1 | 0 (0.00) | 1 (0.45) | 1 (0.89) | 0 (0.00) | 0 (0.00) | - | NA | - | NA | - | NA | - | NA |
| 5′H-71/3′H-3 | 1 (0.45) | 0 (0.00) | 0 (0.00) | 0 (0.00) | 0 (0.00) | - | NA | - | NA | - | NA | - | NA |
| 5′H-72/3′H-3 | 0 (0.00) | 1 (0.45) | 1 (0.89) | 0 (0.00) | 0 (0.00) | - | NA | - | NA | - | NA | - | NA |
| 5′H-72/3′H-8 | 0 (0.00) | 1 (0.45) | 1 (0.89) | 0 (0.00) | 0 (0.00) | - | NA | - | NA | - | NA | - | NA |
| 5′H-73/3′H-1 | 1 (0.45) | 0 (0.00) | 0 (0.00) | 0 (0.00) | 0 (0.00) | - | NA | - | NA | - | NA | - | NA |
| 5′H-42/3′H-2 | 0 (0.00) | 1 (0.45) | 1 (0.89) | 0 (0.00) | 0 (0.00) | - | NA | - | NA | - | NA | - | NA |
| 5′H-43/3′H-1 | 0 (0.00) | 1 (0.45) | 1 (0.89) | 0 (0.00) | 0 (0.00) | - | NA | - | NA | - | NA | - | NA |
| 5′H-11/3′H-2 | 0 (0.00) | 1 (0.45) | 1 (0.89) | 0 (0.00) | 0 (0.00) | - | NA | - | NA | - | NA | - | NA |
| 5′H-52/3′H-1 | 1 (0.45) | 0 (0.00) | 0 (0.00) | 0 (0.00) | 0 (0.00) | - | NA | - | NA | - | NA | - | NA |
| 5′H-44/3′H-3 | 0 (0.00) | 1 (0.45) | 1 (0.89) | 0 (0.00) | 0 (0.00) | - | NA | - | NA | - | NA | - | NA |
| 5′H-20/3′H-1 | 1 (0.45) | 0 (0.00) | 0 (0.00) | 0 (0.00) | 0 (0.00) | - | NA | - | NA | - | NA | - | NA |
| 5′H-74/3′H-2 | 1 (0.45) | 0 (0.00) | 0 (0.00) | 0 (0.00) | 0 (0.00) | - | NA | - | NA | - | NA | - | NA |
| 5′H-75/3′H-1 | 0 (0.00) | 1 (0.45) | 0 (0.00) | 1 (1.78) | 0 (0.00) | - | NA | - | NA | - | NA | - | NA |
| 5′H-46/3′H-3 | 1 (0.45) | 0 (0.00) | 0 (0.00) | 0 (0.00) | 0 (0.00) | - | NA | - | NA | - | NA | - | NA |
| Global p-value for case/control haplotype association was 0.01; NA, not applicable.***** p≤0.05; HTTAXGQB (5′H-60); HGCAYAPA (5′H-61); LTTGXGQB (5′H-62); HGTGYAPA (5′H-63); LGCAYAPA (5′H-64); HTCGYAPA (5′H-65); LTCAYGQB (5′H-66); HTTAXGQA (5′H-67); HTTGXGPA (5′H-68); HGTAYGPA (5′H-69); LTCAYGQA (5′H-70); LTCAXGQB (5′H-71); LTCGXAQB (5′H-72); LGTAYGQB (5′H-73); LGCGYGQB (5′H-74); LGCGXGPA (5′H-75). | | | | | | | | | | | | | |
