# Supplementary material for: Impact of SNPs interplay across the locus of MBL2, between MBL and Dectin-1 gene, on women’s risk of developing recurrent vulvovaginal infections
Source: Cell Biosci. 2019 May 7;9:35. doi: 10.1186/s13578-019-0300-4 (PMC6505208; doi:10.1186/s13578-019-0300-4)
Supplement: Supplementary file 3 — Additional file 3. MBL2 SNPs frequencies in present study population (North Indian) and modern human populations in human genomes variation project i.e. The 1000 Genomes Project (Phase 3). [file 13578_2019_300_MOESM3_ESM.docx]

**Additional file 3:** *MBL2* SNPs frequencies in present study population (North Indian) and modern human populations in human genomes variation project *i.e.* The 1000 Genomes Project (Phase 3) (https://www.ncbi.nlm.nih.gov/variation/tools/1000genomes/).

| **SNPs** | **Present study population** | **The 1000 Genomes Project (Phase 3)** | | | | | |
| --- | --- | --- | --- | --- | --- | --- | --- |
|  | **North Indian**  **(N = 461) ^†^**  **(N = 218) ^‡^** | **Global**  **(N = 2504)** | **AFR**  **(N = 661)** | **AMR**  **(N = 347)** | **EAS**  **(N = 504)** | **EUR**  **(N = 503)** | **SAS**  **(N = 489)** |
| **Additional variants** | | | | | | | |
| **rs11003124** | | | | | | | |
| T | 0.770 | 0.716 | 0.460 | 0.832 | 0.865 | 0.803 | 0.743 |
| G | 0.230 | 0.283 | 0.540 | 0.168 | 0.135 | 0.197 | 0.257 |
| **rs7084554** | | | | | | | |
| **T** | 0.790 | 0.716 | 0.461 | 0.830 | 0.863 | 0.803 | 0.745 |
| **C** | 0.210 | 0.283 | 0.538 | 0.170 | 0.137 | 0.197 | 0.255 |
| **rs36014597** | | | | | | | |
| **A** | 0.750 | 0.716 | 0.461 | 0.828 | 0.863 | 0.8022 | 0.744 |
| **G** | 0.250 | 0.283 | 0.539 | 0.172 | 0.137 | 0.1978 | 0.255 |
| **rs11003123** | | | | | | | |
| **G** | 0.670 | 0.716 | 0.459 | 0.830 | 0.862 | 0.8022 | 0.744 |
| **A** | 0.330 | 0.283 | 0.540 | 0.170 | 0.137 | 0.1978 | 0.255 |
| AFR: African; AMR: Ad Mixed American; EAS: East Asian; EUR: European; SAS: South Asian | | | | | | | |
